# Development of a magnetic molecularly imprinted polymer for the removal of dexamethasone from river nile water

**DOI:** 10.1038/s41598-025-32056-0

**Published:** 2025-12-29

**Authors:** Salma A. Mokbel, Mohammad Abdel-Halim, Mehmet Dinc, Boris Mizaikoff, Nesrine A. El Gohary

**Affiliations:** 1 Pharmaceutical Chemistry Department, Faculty of Pharmacy and Biotechnology, German University in Cairo, Cairo, 11835 Egypt; 2 Department of Pharmaceutical Chemistry, School of Life and Medical Sciences, University of Hertfordshire, Hosted by the Global Academic Foundation, New Administrative Capital, Cairo, Egypt; 3 Hahn-Schickard, 89077 Ulm, Germany; 4 Institute of Analytical and Bioanalytical Chemistry, Ulm University, 89081 Ulm, Germany

**Keywords:** Biochemistry, Chemistry, Environmental sciences, Nanoscience and technology

## Abstract

Magnetic molecularly imprinted polymers (MMIPs) were synthesized using 3-aminophenylboronic acid (APBA) for recognition of dexamethasone (DEX). The particles were characterized by scanning electron microscopy (SEM), transmission electron microscopy (TEM), Brunauer–Emmett–Teller (BET) analysis, Barrett–Joyner–Halenda (BJH) pore size distribution, and X-ray photoelectron spectroscopy (XPS). Kinetic evaluation showed pseudo-first-order (PFO) behavior with rapid uptake within 7 min and equilibrium at 120 min, while Langmuir modeling gave a maximum adsorption capacity of q_max_ = 500.0 µmol g⁻¹. The optimized polymer exhibited 67.0% binding in rebinding experiments. Magnetic solid-phase extraction (MSPE) was conducted using 20 mg sorbent and 3 mL sample, giving 90.08% ± 7.25% recovery in River Nile water with an imprinting factor (IF = 2.21). Greenness evaluation gave a solvent intensity (SI) of 3.5 mL per sample and an analytical method volume intensity (AMVI) of 9.0 mL. EcoScale (~ 89) placed the synthesis in the excellent category, with AGREEmip (0.63) indicating good compliance with green principles, while the MSPE workflow showed favorable greenness (EcoScale = 77; AGREEprep = 0.51). DEX was quantified using a newly developed and validated ultra-performance liquid chromatography–tandem mass spectrometry (UPLC–MS/MS) method. This APBA-based MMIP–MSPE workflow offers a rapid and environmentally compatible approach for DEX monitoring.

**Supplementary Information:**

The online version contains supplementary material available at 10.1038/s41598-025-32056-0.

## Introduction

The Nile River, the longest in the world, spans eleven countries and covers nearly 10% of the African continent. It supports millions of people by providing water for irrigation, hydroelectric power, transportation, fishing, and tourism. Despite its importance, the Nile faces increasing water quality degradation due to nonpoint sources such as agricultural runoff and mining waste^1,2^, It is also affected by point sources such as domestic and industrial discharge, particularly in urban areas. The lack of adequate wastewater treatment facilities further exacerbates the problem, allowing untreated effluents to enter the river. To address this issue, efficient and cost-effective wastewater treatment strategies are urgently required^1^.

In recent years, the environmental occurrence and associated risks of steroid hormones have received considerable attention. Glucocorticoids are a key class of endocrine-disrupting compounds acting through the glucocorticoid signaling pathway. They have been widely detected in aquatic environments^3^. Among them, dexamethasone (DEX) is a synthetic corticosteroid extensively used for its anti-inflammatory and immunosuppressive properties^4,5^. As an endocrine disruptor, it can mimic or interfere with hormonal regulation. This may lead to adverse health effects^6^, including osteoporosis, adrenal suppression, increased fracture risk, reduced thyroid hormone levels, and greater susceptibility to secondary infections^7^.

DEX is considered an emerging contaminant in aquatic systems due to its endocrine-disrupting potential at low concentrations^8^, high production volume, and widespread environmental release. It has been detected in various matrices, including river water, drinking water, surface water, and wastewater, at concentrations ranging from ng L⁻¹ to µg L⁻¹. This widespread occurrence has become more pronounced following its extensive use during the COVID-19 pandemic. Its presence in surface and drinking water highlights its environmental persistence. It also demonstrates the limitations of conventional wastewater treatment systems in removing it. Consequently, there is an urgent need for efficient, sensitive, and cost-effective methods for its detection^9^.

Molecularly imprinted polymers (MIPs) have attracted significant scientific interest due to their high specificity and selectivity, as well as their resistance to harsh environmental conditions, including extreme temperatures^10–12^. They are also known for their excellent stability under high pressure and in acidic, basic, or organic media. This stability enables long-term storage without loss of functionality^13^. Moreover, MIPs are cost-effective to produce and offer advantages such as reusability^14^, flexibility^15^, environmental compatibility^16^, and reversible template binding^17^.

Among the various synthesis methods, surface molecular imprinting is particularly advantageous. It offers numerous accessible recognition sites and provides more uniform and effective binding than traditional bulk imprinting. This facilitates faster and easier template removal. Consequently, surface-imprinted MIPs are widely employed as solid-phase extraction (SPE) sorbents^18^.

Magnetic molecularly imprinted polymers (MMIPs) combine the advantages of molecular imprinting and magnetic separation. Molecular imprinting provides highly specific and selective binding for target analytes, including pesticides, dyes, and drugs^13,19^. In addition, the polymer layer helps prevent aggregation of magnetic nanoparticles^14^. Magnetic separation enables rapid and straightforward recovery using an external magnet. This eliminates the need for filtration or centrifugation, steps often required in traditional MIPs^20^.

Iron oxide (Fe₃O₄) is the most widely used magnetic particle^14^because of its biocompatibility^21^, high coercivity^22^, low toxicity^18^, the large surface area, ease of modification, and strong magnetic susceptibility. It is the most magnetic of all naturally occurring minerals^17^. Its surface is rich in hydroxyl groups^23^, enabling functionalization with active groups such as –COOH, –OH, and –NH₂^24^.

Molecularly imprinted magnetic solid-phase extraction (MI-MSPE), which combines the benefits of molecularly imprinted SPE and magnetic SPE, offers high selectivity, strong adsorption capacity, and simplified separation^25^. It also reduces time and reagent use^14^, supports greener solvents^26^, and enhances environmental sustainability^27,28^.

3-aminophenylboronic acid (APBA), a boronic acid derivative, binds reversibly with cis-diols to form cyclic esters. This enables selective molecular recognition^29–31^. Its high biocompatibility and unique chemistry make it valuable in separation, sensing, and self-assembly.

Over the past decade, several sorbent-based strategies have been investigated for DEX removal, including activated carbon, C18 SPE cartridges, and modified clays. For example, Sulaiman et al. demonstrated that micelle–clay composites achieved higher removal efficiency than activated carbon when applied to wastewater. This confirmed the potential of sorbent-based materials for DEX remediation. Building on this, MIPs offer an alternative approach, in which synthetic recognition sites can be tailored around a target molecule, enabling more robust and application-specific extraction^32^.

Within this context, eleven different MIP systems targeting DEX have been reported. Notably, only four employed magnetite-based supports. All were restricted to cosmetic or model matrices, and none were validated in environmental waters^4,33–35^. The remaining studies described non-magnetic MIPs in aqueous solutions^36^, MIP–aptamer sensors in natural water^37^, or biomedical controlled-release systems^38^. Taken together, these reports demonstrate the feasibility of DEX imprinting. However, they also highlight a critical gap: no magnetite-based MMIP has been applied to real environmental waters. Furthermore, none have utilized APBA as a functional monomer, despite its unique ability to form reversible covalent interactions with DEX.

To address this gap, an APBA-functionalized Fe₃O₄@SiO₂ MMIP is introduced as an additional design route for DEX imprinting. APBA exploits the cis-diol motif in DEX via boronate–diol complexation instead of relying solely on non-covalent interactions used in earlier systems. The formulation integrates surface imprinting with magnetic handling enabling rapid adsorption, easy handling, and centrifugation-free separation. The workflow is designed to align with green-chemistry principles. The developed APBA-based MMIP was coupled with a newly developed and validated ultra-performance liquid chromatography coupled with tandem mass spectrometry (UPLC–MS/MS) method for the analysis of DEX in River Nile water. Adsorption kinetics and isotherm modelling are carried out to provide mechanistic insight into the binding process. The analytical performance lies within the ranges reported for earlier DEX-MIPs (Table 1) while using a distinct imprinting chemistry and workflow. The method’s environmental profile is additionally assessed using, Eco-Scale, AGREEprep and AGREEmip as part of the greenness evaluation.

**Table 1:** Comparison of different MIPs reported for dexamethasone DEX analysis.

Parameter	Liu et al.^33^	Du et al.^4^	Hang et al.^34^	Adauto et al.^36^	This study (MMIP10)
Magnetic Core	Yes	Yes	Yes	No	Yes
Monomer	Methacrylic acid	Methacrylic acid	DES-derived monomer	2-Hydroxyethyl methacrylate	APBA
Porogen	Methanol	Methanol	Deep eutectic solvent	ACN	Phosphate buffer
Binding Solvent	ACN/water	ACN/water	ACN	Ethanol-based aqueous	Ultrapure water (pH 10)
Binding Onset (min)	5	5	5	5	7
Binding Equilibrium (min)	30	180	120	180	120
Recovery (%)	91.2–104.3	93.8–97.6	81–97	65.6–71.8	90.1 ± 7.3
Sample Type	Cosmetics (lotion, toner, mask)	Cosmetics	Cosmetics (lotions/creams)	Drinking & river water	River Nile water
Magnetic separation	Yes	Yes	Yes	No	Yes
AGREEmip Score	0.5	0.67	0.62	0.61	0.63

## Experimental

### Materials and chemicals

Absolute ethanol (analytical grade), acetonitrile (ACN, HPLC grade), ammonium persulfate (≥ 98%), formic acid (≥ 98%), glutaraldehyde (GA, 25% aqueous solution), magnetite nanoparticles (Fe₃O₄, 50–100 nm), methanol (HPLC grade), potassium chloride (analytical grade), sodium bicarbonate (analytical grade), sodium carbonate (analytical grade), sodium chloride (analytical grade), sodium hydroxide pellets (analytical grade), sodium phosphate dibasic dihydrate (analytical grade), sodium phosphate monobasic monohydrate (analytical grade), triamcinolone acetonide (TCA) (≥ 98%), and tetraethyl orthosilicate (TEOS, ≥ 98%) were obtained from Sigma-Aldrich (Germany).

APBA (97%), (3-aminopropyl)triethoxysilane (APTES, 99%), DEX (≥ 98%), ninhydrin (≥ 98%), potassium phosphate monobasic (KH₂PO₄, analytical grade), and sodium borohydride (NaBH₄, ≥ 98%) were obtained from Alfa Aesar (Germany).

Ammonia solution (25% and 33%), glacial acetic acid (AcOH, ≥ 99.7%), and hydrochloric acid (37%) were obtained from Fluka (Germany). Betamethasone (BTZ, ≥ 98%) was obtained from Acros Organics (Germany). Ultrapure water (UPW, resistivity 18.2 MΩ·cm) was produced using a Purelab UHQ system (ELGA, United Kingdom).

### Synthesis and binding evaluation

Only MMIP10 and MMIP11 are discussed in this work, as they demonstrated the most promising characteristics during preliminary assessment.

### Amination of magnetic particles (Fe_3_O_4_@SiO_2_–NH_2_)

A total of 0.2 g of magnetite was dispersed in 250 mL of ethanol and ultrasonicated for 20 min. Subsequently, 4 mL of 25% ammonia solution and 3 mL of UPW were added. This was followed by the addition of 0.6 mL of APTES (2.56 mmol**)** and 0.2 mL of TEOS (0.90 mmol**)**. The mixture was mechanically stirred at 300 rpm for 24 h at room temperature (25 °C). The resulting particles were washed four times with 100 mL of UPW and twice with 50 mL of methanol, then dried overnight at room temperature (25 °C). To confirm the presence of amino groups on the surface of the Fe₃O₄@SiO₂–NH₂ particles, a ninhydrin test was performed. Briefly, 1 mL of ninhydrin solution, prepared by dissolving 0.1 g of ninhydrin in 50 mL of ethanol **(**2 mg mL⁻¹**)**, was added to 0.01 g of the synthesized particles. The mixture was incubated in an Eppendorf tube at 100 °C for 1 h.

### Immobilization by imine formation Fe_3_O_4_ @SiO_2_–NH_2_@GA@APBA

The synthesis was adapted from Lin et al. (2011) with slight modifications^39^. Initially, 0.25 g of Fe₃O₄@SiO₂–NH₂ was suspended in 50 mL of PBS **(**0.1 M, pH 7.4**)** and ultrasonicated for 20 min. Subsequently, 10 mL of 25% GA was added. The mixture was then stirred for 6 h. The resulting particles were separated using a strong magnet and washed three times with 50 mL of PBS.

In a separate beaker, 0.5 g of APBA (3.62 mmol) was dissolved in 50 mL of PBS and then added to the washed particles. The mixture was ultrasonicated for 10 min, followed by mechanical stirring for an additional 3 h at 300 rpm at room temperature (25 °C). To reduce the imine (C = N) bonds to more stable amine (C–N) linkages, thereby enhancing the stability of the immobilized GA, 0.2 g of sodium borohydride (5.29 mmol) was slowly added. It was then stirred for 3 h under the same conditions.

The particles were then magnetically separated and washed three times with 100 mL of UPW and three times with 100 mL of ethanol. Finally, the particles were air-dried at room temperature (25 °C) for subsequent use. Successful immobilization was confirmed via a ninhydrin test, performed as described previously.

### MMIP preparation using Fe_3_O_4_@SiO_2_–NH_2_@GA@APBA

MMIP10 was synthesized as follows: 0.1 g of Fe₃O₄@SiO₂–NH₂@GA@APBA was suspended in 125 mL of phosphate buffer (0.073 M, pH 7.2) in a 250 mL beaker, followed by the addition of 0.342 g of APBA (2.48 mmol). The mixture was ultrasonicated for 10 min. Simultaneously, 0.196 g of DEX (0.50 mmol) was dissolved in 70 mL of ACN, and 0.342 g of APBA **(**2.48 mmol; yielding a final concentration of 20 mM**)** dissolved in 60 mL of phosphate buffer (0.073 M, pH 7.2) was then added to a separate beaker. Both solutions were stirred for 30 min before the contents of the second beaker were added to the first. This step resulted in a template-to-monomer molar ratio of DEX: APBA = 1:5. The resulting mixture was allowed to undergo prepolymerization assembly for 13 h. Polymerization of APBA was then initiated by the dropwise addition of 13 mL of freshly prepared ammonium persulfate solution **(**(NH₄)₂S₂O₈, 100 mM). The polymerization proceeded for 24 h at 60 °C with stirring at 300 rpm^40^.

The particles were separated using a strong magnet and washed three times with 100 mL of UPW followed by three washes with 100 mL of ethanol. Subsequently, the polymers were subjected to successive washes with 20 mL of a methanol and AcOH mixture (9:1 v/v)^41^. This was followed by three additional washes with pure methanol for 15 min each. The extraction process continued until no DEX was detected in the filtrate by UPLC–MS/MS.

Based on Basan et al. (2018)^42^ and Li et al. (2009),^43^ the polymerization reaction can proceed more rapidly without heating. Accordingly, MMIP11 was prepared following the same procedure, except that the prepolymerization assembly lasted only 1 h, and polymerization was conducted for 2 h at room temperature (25 °C). For each MMIP, a corresponding magnetic nonimprinted polymer (MNIP) was prepared under identical conditions but in the absence of the template.

### Batch equilibrium rebinding studies

All binding experiments were conducted in triplicate (*n* = 3) using independent polymer and analyte solutions, and results are reported as mean ± standard deviation (SD). Batch rebinding studies for MMIP10, MMIP11, and their corresponding MNIPs were conducted to evaluate the performance of the synthesized polymers. In these studies, 10 mg of MMIP10 was incubated in a 5 mL Eppendorf tube containing 3 mL of DEX solution (5.0 × 10⁻⁵ M) prepared in various solvents, including ACN; UPW at pH 6.0, 8.0, 9.0, 10.0, and 12.0, with pH adjusted directly using 0.1 M NaOH or 0.1 M HCl. For MMIP11, the rebinding studies were carried out over 2 h using a DEX solution (5.0 × 10⁻⁵ M) prepared in UPW at pH 6.0.

The amount of DEX bound to the polymers was calculated by subtracting the concentration of free, unbound DEX from the initial concentration at each tested condition^44^. The binding capacities (B, µmol g⁻¹) of the MMIPs and MNIPs were calculated using Eq. (9)^45^.

1 $$\:\:\:\:\:\:\:\:\:\:\:\:\:\:\:B=\frac{\left({C}_{i}-{C}_{f}\right)x\:Vx1000}{M}$$

Where C_i_ is the initial solution concentration (mM), C_f_is the free concentration of DEX after adsorption (mM), V is the volume of the sample solution tested (mL) and M is the mass of polymer used (mg). The binding capacities of the MMIPs were compared with those of their corresponding MNIPs to determine the IF according to Eq. (2)^45^.

2 $$\:\:\:\:\:\:\:\:\:\:IF=\frac{{Q}_{MIP}}{{Q}_{NIP}}$$

### Characterization

The morphology and composition of the particles were analyzed using a Quanta 3D FEG scanning electron microscope (SEM) (FEI Company, Eindhoven, Netherlands). To assess the size and thickness of the core–shell film, transmission electron microscopy (TEM) was performed using a Zeiss EM 10 instrument (Oberkochen, Germany). Nitrogen adsorption/desorption isotherms were measured with a Quadrasorb Station SI (Quantachrome GmbH & Co. KG) after degassing the samples at 77.3 K under vacuum for 3 h. The specific surface area was determined using the Brunauer–Emmett–Teller (BET) method, while the pore volume and pore size were calculated using the Barrett–Joyner–Halenda (BJH) method. Surface elemental composition was evaluated by X-ray photoelectron spectroscopy (XPS) using a PHI 5800 ESCA system (Physical Electronics, USA).

### Binding isotherms

To investigate the binding isotherms of the most efficient MMIP (MMIP10), a series of DEX solutions at nine different concentrations (5.0 × 10⁻⁶, 7.5 × 10⁻⁶, 1.0 × 10⁻⁵, 2.5 × 10⁻⁵, 5.0 × 10⁻⁵, 7.5 × 10⁻⁵, 2.5 × 10⁻⁴, 5.0 × 10⁻⁴, and 7.5 × 10⁻⁴ M) were prepared in carbonate buffer (0.1 M, pH 10.0). Each concentration point was measured in triplicate (*n* = 3), and results are reported as mean ± SD. For each concentration, 10 mg of MMIP10 was incubated with 3 mL of DEX solution under shaking conditions for 2 h at room temperature (25 °C). Following incubation, the polymer particles were separated using a strong magnet, and the supernatant was filtered through a 0.22 μm Whatman syringe filter. The residual concentration of DEX in the clear supernatant was quantified using UPLC–MS/MS. The amount of DEX bound to the polymer was then calculated as previously described. A binding isotherm was constructed by plotting the amount of DEX bound (B) against the initial DEX concentration. The resulting data were further analyzed using both Langmuir and Freundlich isotherm models to estimate the binding parameters of MMIP10. The log form of the Freundlich isotherm was applied, as shown in (Eq. 3)^46^.

3 $$\:log\:{q}_{e}\:=log\:{K}_{F}\:+\frac{1}{n}\cdot\:log\:{c}_{e}$$

where q_e_ is the amount of DEX adsorbed in µmol g⁻¹, C_e_ is the equilibrium concentration of DEX in solution in µM, and K_F_ and n are Freundlich constants, where K_F_ is a measure of the adsorption capacity of the sorbent and where 1/n is the heterogeneity factor ranging from 0 to 1. As the value approaches 1, the heterogeneity decreases, with a completely homogeneous system at a value of 1.

The linear form of the Langmuir isotherm was used (Eq. 4)^47^

4 $$\:\frac{1}{{q}_{e}}=\frac{1}{\left({K}_{L\cdot\:}{q}_{max}\right)}\cdot\:\frac{1}{{c}_{e}}+\frac{1}{{q}_{max}}$$

where q_e_ is the amount of DEX adsorbed in µmol g⁻¹, C_e_ is the equilibrium concentration of DEX in solution in µM, K_L_ is the Langmuir constant in L µM⁻¹, and q_max_ is the maximum number of binding sites in µmol g⁻¹.

### Binding kinetics

To optimize the binding conditions, 3 mL of a 5.0 × 10⁻⁵ M DEX solution in carbonate buffer (0.1 M, pH 10.0) was added to separate 5 mL Eppendorf tubes, each containing 10 mg of MMIP10. Each tube was incubated for a different time interval (7, 15, 60, 120, 150, and 180 min) under constant shaking to assess the effect of contact time on DEX binding. Each time condition was tested in triplicate (*n* = 3), and results are reported as mean ± SD. After incubation, the polymer particles were magnetically separated, and the supernatant was filtered through a 0.22 μm Whatman syringe filter. The residual concentration of DEX in the filtrate was determined using the UPLC–MS/MS method. The amount of DEX bound to the polymer, along with the IF, was calculated using Eqs. 1 and 2.

To further analyze the adsorption process, the kinetic data were fitted to the pseudo-first-order (PFO) and pseudo-second-order (PSO) kinetic models, expressed by Eqs. 5^48^ and 6^49^, respectively

5 $$\:ln\left({q}_{e}-\:{q}_{t}\right)=\:ln\:{q}_{e}-\:{k}_{1}$$

6 $$\:\frac{t}{{q}_{t}}=\frac{1}{{k}_{2}{{q}_{e}}^{2}}+{\frac{t}{q}}_{e}$$.

where q_t_ (µmol g⁻¹) is the amount of DEX adsorbed at time t, q_e_ (µmol g⁻¹) is the adsorption capacity at equilibrium, and k_1_ (min⁻¹) and k_2_ (g µmol⁻¹ min⁻¹) are the rate constants for the PFO and PSO models, respectively.

### Selectivity studies

The ability of MMIP10 to predominantly bind and retain (DEX, C₂₂H₂₉FO₅) rather than its isomer was studied. BTZ (C₂₂H₂₉FO₅) was evaluated using a noncompetitive binding study. In this experiment, 3 mL of a 5.0 × 10⁻⁵ M BTZ solution prepared in carbonate buffer (0.1 M, pH 10.0) was added to 10 mg of MMIP10 and its corresponding MNIP, and the mixture was incubated under shaking in an Eppendorf shaker. The time points of 7 min and 2 h were selected based on preliminary binding kinetics experiments (Figure S7B), representing the rapid initial adsorption phase and the equilibrium binding state, respectively. Two parallel studies were then conducted at these incubation periods. Both 7-min and 2-h selectivity experiments were performed in triplicate (*n* = 3), and the results are reported as mean ± SD. After incubation, the polymer particles were magnetically separated, and the supernatant was filtered through a 0.22 μm Whatman syringe filter. The residual concentration of BTZ in the clear supernatant was quantified using the UPLC–MS/MS method.

The selectivity factor (α) was calculated according to Eqs. 7 and 8.

7 $$\:\:\alpha\:=\frac{{Q}_{analyte}}{{Q}_{analogue}}$$

8 $$\:\alpha\:=\frac{{IF}_{analyte}}{{IF\:}_{analogue}}$$

where *Q*_analyte_ is the amount of analyte bound by the MIP, *Q*_analogue_ is the amount of structural analog bound by the MIP, IF_analyte_ is the imprinting factor of the analyte, and IF_analogue_ is the imprinting factor of the analog^45^.
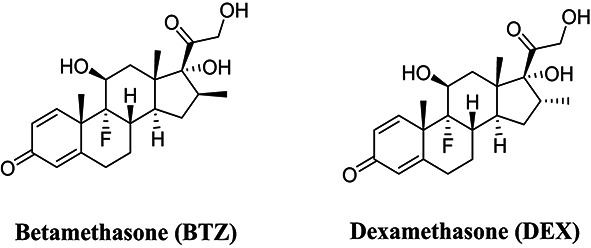


### UPLC–MS/MS method development

A new UPLC–MS/MS method was developed to quantify DEX extracted from different matrices. In this newly developed method, TCA (2.3 × 10⁻⁶ M) was used as the internal standard due to its structural similarity to DEX, comparable retention and ESI(+) response, and a distinct multiple reaction monitoring (MRM) transition (m/z 435.2 → 415.2) with no overlap. A constant volume of the IS solution was added to all samples prior to analysis. A DEX stock solution (1.00 × 10^− 2^ M) was prepared in ACN, and proper dilutions in carbonate buffer (0.1 M, pH 10.0) were prepared to obtain nine standard solutions of the calibration curve covering the range of 5.0 × 10⁻⁷–1.0 × 10⁻⁴ M.

The UPLC–MS/MS experiments were carried out under the following conditions: gradient elution at a flow rate of 0.5 mL min⁻¹ was conducted for chromatographic separation by the addition of 0.1% formic acid in water (A) and 0.1% formic acid in ACN (B). The gradient was run as follows: 0 min, 60% A, 40% B; 0.5 min, 60% A, 40% B; 2.5 min, 5% A, 95% B; 3 min, 5% A, 95% B; 3.5 min, 60% A, 40% B; and 4 min, 60% A, 40% B. The injection volume was 10 µL, which was performed using partial loop injection with needle overfill as the injection technique. The column temperature was set at 40 °C. The ESI source was operated in positive mode. Nitrogen was used as the desolvation and cone gas at flow rates of 1000 and 20 L h⁻¹, respectively. Argon was used as the collision gas at a pressure of approximately 3.67 × 10⁻³ mbar. The optimal MS parameters were as follows: capillary voltage, 3.06 kV; radio frequency (RF) lens voltage, 2.5 V; source temperature, 150 °C; and desolvation gas temperature, 500 °C. Cone voltages of 20 and 30 V were used for DEX and TCA, respectively.

Quantification was performed via MRM of the transitions of m/z 393.1 > 373.19 with a collision energy of 10 V for DEX and m/z 435.178 > 415.209 with a collision energy of 8 V for TCA. The dwell time was automatically set by MassLynx.

### UPLC–MS/MS method validation

In accordance with International Council for Harmonisation (ICH) guidelines, the developed UPLC–MS/MS method was validated for linearity, limit of detection (LOD), limit of quantification (LOQ), precision (intra- and interday), and accuracy. For linearity assessment, a fixed volume of 2.3 × 10⁻⁶ M TCA was added as an internal standard to each sample prior to analysis. A calibration curve was constructed over the concentration range of 5.0 × 10⁻⁷ to 1.00 × 10⁻⁴ M by plotting the peak area ratio of DEX/TCA versus the corresponding DEX concentrations. Each concentration was analyzed in triplicate. Linearity was evaluated using the squared regression coefficient (R²). The LOD and LOQ were calculated based on the calibration curve using Eqs. 9 and 10, respectively.

9 $$\:LOD=3.3\:\left(\frac{\sigma\:}{S}\right)\:\:\:\:\:\:\:\:\:$$

10 $$\:\:LOQ\:=10\:\left(\frac{\sigma\:}{S}\right)\:\:\:\:\:\:\:\:\:$$

where “σ” is the standard deviation of the intercept and S is the slope of the calibration curve^50^.

Precision reflects the repeatability and reproducibility of an analytical method and was evaluated in terms of intra- and interday precision, expressed as relative standard deviation (RSD). Intraday precision was assessed by analyzing nine standard DEX solutions (5.0 × 10⁻⁷, 1.0 × 10⁻⁶, 5.0 × 10⁻⁶, 1.5 × 10⁻⁵, 3.5 × 10⁻⁵, 5.0 × 10⁻⁵, 6.5 × 10⁻⁵, 7.5 × 10⁻⁵, and 1.0 × 10⁻⁴ M) three times within the same day. For interday precision, the calibration curve was repeated on three separate days^51^.

Accuracy was determined by comparing the measured concentrations obtained using the UPLC–MS/MS method with the corresponding theoretical concentrations of four standard solutions selected from the calibration range. The results are reported as the mean recovery percentage and RSD, based on three replicate measurements for each concentration^52^.

### Application of MMIP10 in MI-MSPE of DEX from nile river water samples

In this study, MMIP10 was used as the selective sorbent for magnetic SPE. The extraction process consisted of three main steps: sample loading, removal of interferents by washing, and elution of the analyte using an appropriate solvent. Carbonate buffer (0.1 M, pH 10.0), prepared using undiluted tap water collected from the 5th Settlement in New Cairo City, Cairo Governorate (sourced directly from the Nile River), was used as the loading solvent (Figure S12).

A volume of 3 mL of DEX solution (5.0 × 10⁻⁵ M) in carbonate buffer was added to 5 mL Eppendorf tubes containing 20 mg of the polymer, and the mixtures were incubated under shaking for 2 h. After incubation, the polymer particles were washed with 2 mL of UPW for 30 min to remove nonspecifically bound interferents. Elution of the bound DEX was then performed using 3 mL of AcOH in methanol at room temperature (25 °C) for 2 h. Four concentrations of AcOH in methanol (5%, 10%, 15%, and 20% v/v) were tested to determine the optimal elution conditions. Each extraction condition was evaluated in triplicate (*n* = 3) using independent sample tubes, and the results are reported as mean ± SD.

Following elution, the solvent was evaporated using a concentrator, and the residue was reconstituted in carbonate buffer (pH 10.0) with the addition of 0.5 mL ACN to facilitate DEX solubilization. The solution was filtered through a 0.22 μm Whatman syringe filter and analyzed by the UPLC–MS/MS method. The recovery percentage of DEX was then calculated.

### Assessment of method greenness

Green analytical chemistry aims to minimize the use of hazardous reagents, reduce waste, and improve the sustainability of analytical procedures. To evaluate the greenness of the developed method, two volume-based indicators were first calculated. Solvent Intensity (SI) reflects the total volume of organic solvent consumed per analysis. Analytical Method Volume Intensity (AMVI) considers both organic and aqueous solvents. In this calculation, water is excluded from SI but included in AMVI. This provides a broader picture of solvent usage^53^.

The greenness of the MMIP10 synthesis was assessed using the EcoScale framework. The evaluation was performed using the web-based calculator. All penalty-point inputs were cross-checked against the actual experimental conditions. The SDS data of the reagents employed were also consulted^54^.

To evaluate the greenness of the SPE stage, a comparative analysis was performed. The present MMIP-based SPE protocol was compared with two literature SPE methods based on conventional sorbents a conventional silica-based C18 sorbent^55^, and a Polymeric HLB sorbent (Oasis HLB)^56^. This comparison employed AGREEprep and a full EcoScale evaluation of the SPE workflows. Penalties were assigned for reagents, hazards, solvent volume, energy, waste, and instrumentation^54,57^.

Finally, the overall imprinting-based analytical strategy was evaluated using AGREEmip. This metric extends AGREE by incorporating molecular-imprinting-specific design and operational criteria^58^. AGREEmip was applied to the present method. It was also applied to four previously reported MIP-based extraction protocols for DEX. This enabled a holistic comparison with existing imprinting approaches.

EcoScale: ≥75 excellent; 50–74 acceptable; <50 inadequate^54^.

AGREE-type metrics AGREEprep and AGREEmip provide 0–1 score that reflect the extent of alignment with green chemistry principles, with higher values indicating better greenness^57,58^.

## Results and discussion

### Synthesis of MMIPs and batch equilibrium rebinding studies

#### Amination of magnetite and covalent immobilization of boronic acid on Fe_3_O_4_ @SiO_2_–NH_2_

An immobilization strategy was implemented, which has also been reported to enhance adsorption capacity^18^. Initially, amine functionalities were added to the surface of magnetite nanoparticles using APTES and TEOS. These surface amine groups were subsequently covalently linked to APBA prior to polymerization. Successful amination was confirmed by the ninhydrin assay, which yielded a characteristic violet coloration indicative of primary amine groups, as illustrated in Figure S1.

Subsequent functionalization involved imine bond formation, as illustrated in Figure S2. The reaction started by reacting the aminated magnetite with GA. This formed an imine bond between the carbonyl group of the GA and the amino group of Fe_3_O_4_@ SiO_2_–NH_2_. This was followed by the addition of APBA, which reacted with the remaining free aldehyde groups of GA to form additional imine bonds. Finally, the imine linkages (C = N) were reduced to more stable secondary amines (C–N) using sodium borohydride. Importantly, sodium borohydride was introduced only at the final step to prevent premature reduction of the GA carbonyl groups.

The success of the immobilization was further confirmed by a negative result in the ninhydrin test, indicating the absence of unreacted free amine groups, as shown in Figure S1. The resulting functionalized particles were thus deemed suitable for use in the subsequent polymerization step.

### MMIP Preparation using Fe_3_O_4_@SiO_2_–NH_2_@GA@APBA

Prior to polymerization, Fe₃O₄@SiO₂–NH₂@GA@APBA particles were suspended in phosphate buffer (0.073 M, pH 7.2), followed by the addition of 0.342 g of APBA. This step allowed the free APBA to interact with the surface-immobilized APBA, enhancing functional monomer alignment. After a predetermined time, a solution of DEX mixed with APBA was added for prepolymerization. Polymerization was then initiated by the addition of ammonium persulfate.

Literature reports show considerable variation in MMIP synthesis using APBA, primarily in the durations of the prepolymerization and polymerization steps, as well as in the reaction temperature. Based on this, MMIP10 and MMIP11, along with their corresponding MNIPs, were synthesized using identical quantities of core particles, template, and monomer, but under different polymerization conditions. MMIP10 was prepared under harsher conditions—12 h of prepolymerization, 24 h of polymerization, and a reaction temperature of 60 °C^40^, while MMIP11 followed a simplified protocol with 30 min of prepolymerization, 2 h of polymerization, and synthesis at room temperature (25 °C)^42,43^.

In both cases, the polymerization medium turned red, indicating successful polymer formation, as shown in Figure S3. This color change is attributed to the Schiff base (–CH = N–) linkages illustrated in Figure S2. These linkages are formed when the amino group of APBA reacts with the aldehyde group of GA. The resulting Schiff bases act as π-conjugated bridges between the aromatic APBA units and the aldehyde-derived fragments, creating donor–acceptor interactions. This extended conjugation facilitates intramolecular charge transfer, lowering the HOMO–LUMO energy gap and shifting absorption into the visible region. This explains the characteristic red coloration observed during the reaction^59^.

Notably, MMIP10 yielded a darker, more intense red coloration, suggesting a higher polymer content, which was corroborated by XPS and TEM analyses (discussed in the following section). Template removal was performed using a methanol/AcOH mixture (9:1, v/v), in which AcOH disrupted the interactions between the functional monomer and DEX, while methanol facilitated the dissolution and extraction of the template. The process was continued until no detectable DEX remained, as confirmed by UPLC–MS/MS analysis. Residual AcOH was eliminated through subsequent washing with pure methanol.

### Batch equilibrium rebinding studies

**Fig. 1:**
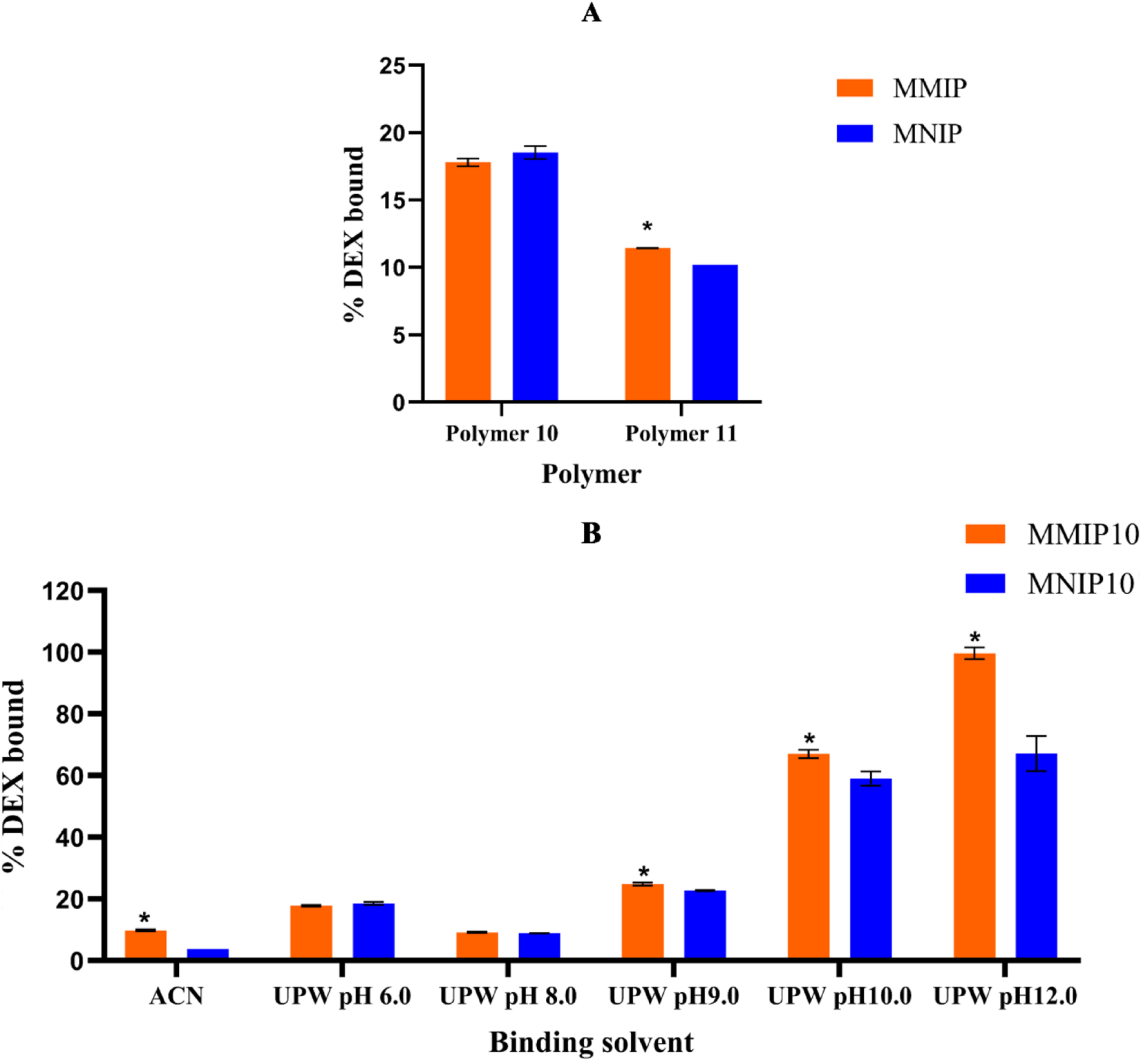
(**A**) Percentage of dexamethasone (DEX) bound to MMIP10, MNIP10, MMIP11, and MNIP11 in ultrapure water (UPW, pH 6.0), expressed relative to the initial DEX concentration of 5.0 × 10⁻⁵ M. (**B**) Effect of solvent and pH (acetonitrile (ACN) and UPW at pH 6.0, 8.0, 9.0, 10.0, and 12.0) on the binding of MMIP10 and MNIP10, expressed relative to the same initial concentration. In each experiment, 10 mg of polymer was incubated with 3 mL of DEX solution (5.0 × 10⁻⁵ M) for 2 h at 25 °C, using either UPW or ACN at the indicated pH values; 3 mL of sample was used in all experiments. An asterisk (*) indicates a statistically significant difference between each MMIP and its corresponding MNIP (unpaired two-tailed t-test, *p* < 0.05). Data represent mean ± standard deviation (SD; *n* = 3).

Both MMIP10 and MMIP11 were evaluated under identical binding conditions. In each experiment, 10 mg of polymer was incubated with 5.0 × 10⁻⁵ M DEX prepared in UPW at pH 6.0 for 2 h. As shown in Fig. 1A, MMIP10 exhibited superior binding performance. It adsorbed 17.80% ± 0.29 (RSD = 1.65%) of the initial DEX concentration, whereas MMIP11 bound only 11.44% ± 0.01 (RSD = 0.06%). This difference was statistically significant (unpaired, two-tailed t test, *p* = 0.0007; significance defined as *p* < 0.05). Likewise, the corresponding non-imprinted polymers differed significantly (MNIP10: 18.53% ± 0.07 vs. MNIP11: 10.20% ± 0.0004; *p* = 0.0010). Based on these results, MMIP10 was selected for subsequent studies.

Boronic acids and their derivatives are weak Lewis acids that exist in equilibrium between an uncharged trigonal form and a charged tetrahedral form. These species form reversible covalent B–O bonds with cis-1,2- and cis-1,3-diol groups, yielding cyclic boronate esters. The uncharged trigonal form is prone to hydrolysis, whereas the charged tetrahedral form stabilizes the boronate ester complex. Phenylboronic acid, for instance, has a pK_a_ of 8.8, allowing it to exist predominantly in the charged form under mildly alkaline conditions^60,61^.

In the presence of Lewis bases, such as hydroxyl groups, interconversion from the sp²-hybridized trigonal form to the sp³-hybridized tetrahedral form occurs readily. Figure S4 illustrates this mechanism. in aqueous media, water reversibly adds to the neutral trigonal boronic acid (Form I) yielding the tetrahedral anionic species (Form II), and releasing a proton. At alkaline pH (pH > 10.0), this tetrahedral form reacts with diols to form stable cyclic boronate esters (Form IV). The general affinity of boronic acids for diols follows the order cis-1,2-diol > cis-1,3-diol > trans-1,2-diol. At neutral pH, however, boronic acids predominantly exist in the trigonal form. These results in unstable intermediates (Form III) that are prone to hydrolysis. Thus, higher pH conditions favor the formation of stable cyclic boronate esters (Form IV), whereas lowering the pH reverses the interaction, releasing the bound diol-containing analyte^62^.

DEX contains hydroxyl groups at positions 17 and 21, which structurally resemble a 1,3-diol moiety. Accordingly, when APBA was used as the functional monomer and the pH was adjusted above its pK_a,_ a significant increase in DEX binding was anticipated. As presented in Fig. 1B, at pH 10.0 and pH 12.0, MMIP10 exhibited markedly enhanced binding capacities, reaching 10.05 µmol g⁻¹ (67.00% ± 1.34, RSD = 2.0%) and 14.95 µmol g⁻¹ (99.66% ± 1.89, RSD = 1.9%), respectively. However, at pH 12.0, significant polymer leaching was observed, as evidenced by the brown coloration of the rebinding solution (Figure S5). In contrast, the solution at pH 10.0 remained nearly clear. Consequently, pH 10.0 was selected as the optimal condition for the rebinding experiments.

Reproducibility across these studies was generally high, with RSD values typically < 4%. A higher RSD (8.51%) was observed for MNIP10 at pH 12.0, which can be attributed to polymer leaching under strongly alkaline conditions. This instability explains the reduced consistency of results at pH 12.0.

Beyond boronate esters, DEX polar groups support hydrogen bonding. Hydroxyls at C11, C17, and C21 and carbonyls at C3 and C20 can act as donors or acceptors. Such interactions have been reported for boronic acid–diol complexes^63^. They have also been confirmed in cyclodextrin inclusion systems, where Fourier Transform Infrared Spectroscopy shifts in DEX hydroxyl and carbonyl bands indicate hydrogen bonding. Crystallographic studies of the glucocorticoid receptor likewise show DEX forming H-bonds via C3 = O and C11–OH^64^.

The nonpolar steroid backbone of DEX further contributes to stabilization. Phenylboronic acid systems benefit from hydrophobic contacts as secondary forces^65^. In the MMIP, the APBA phenyl ring and polymer matrix provide a hydrophobic microenvironment that interacts with the steroid framework. Cyclodextrin studies similarly confirm van der Waals and hydrophobic contacts in stabilizing DEX complexes^64^.

Together, boronate ester formation at C17/C21, hydrogen bonding through hydroxyl and carbonyl groups, and hydrophobic stabilization of the steroid backbone act cooperatively. These mechanisms explain the higher affinity and selectivity of MMIP10 compared with the non-imprinted control (Figure S6).

### Characterization

**Fig. 2:**
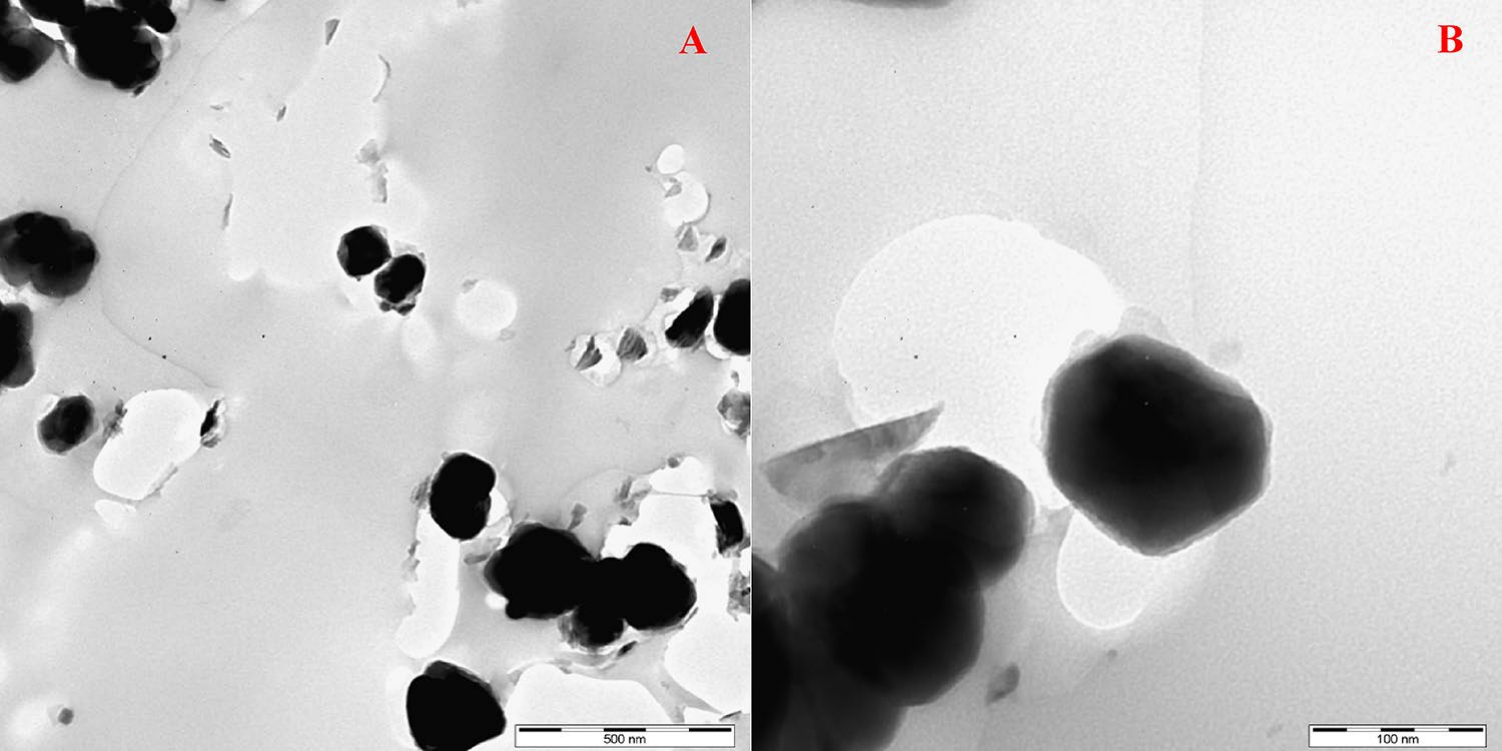
Transmission electron microscopy (TEM) images of (**A**) Fe₃O₄@SiO₂–NH₂ core–shell particles and (**B**) Fe₃O₄@SiO₂–NH₂@GA@APBA after surface functionalization with glutaraldehyde (GA) and 3-aminophenylboronic acid (APBA). A distinct outer layer becomes visible in (**B**), confirming successful grafting of the organic modifiers, while the overall core–shell morphology is preserved.

The surface morphologies of Fe₃O₄@SiO₂**–**NH₂ and Fe₃O₄@SiO₂**–**NH₂@GA@APBA were examined by TEM, as shown in Fig. 2A and B respectively. Based on the TEM images, film thicknesses were calculated. The majority of particles exhibited a spherical shape, although a fraction appeared irregular with a broad size distribution. As shown in Fig. 2B, a distinct polymer film with an average thickness of 8.10 ± 3.39 nm was observed surrounding the Fe₃O₄@SiO₂–NH₂@GA@APBA particles. No such layer was detected in the Fe₃O₄@SiO₂-NH₂ sample (Fig. 2A), confirming successful surface immobilization. This thickness is substantially greater than the 0.5–3 nm layers typically reported for APTES functionalization on Fe₃O₄@SiO₂ surfaces. Therefore, the observed layer represents polymer deposition rather than simple silane grafting^66^. At the same time, the coating is thinner than the 38–100 nm polymer shells reported in other surface-imprinted systems. This suggests that the controlled conditions used here favored the formation of an ultrathin and uniform polymer film^67^. Such thin films are advantageous because they provide sufficient recognition sites while reducing diffusion barriers^68^.

Further evidence of successful APBA immobilization was obtained by XPS. The survey spectrum of Fe₃O₄@SiO₂–NH₂@GA@APBA (Fig. 4) showed the expected Fe 2p (~ 711 eV), O 1 s (~ 531 eV), and Si 2p (~ 103 eV) peaks corresponding to the magnetite core and silica shell, together with pronounced C 1 s (~ 285 eV) and N 1 s (~ 399 eV) signals from the APTES–GA interface. Importantly, a distinct B 1 s peak at ~ 191 eV was observed, indicating the incorporation of APBA onto the particle surface. This observation is consistent with Basan et al. (2018)^42^, who also employed APBA as a functional monomer and validated its successful immobilization through the appearance of characteristic B 1 s and N 1 s signals in survey spectra.

**Fig. 3:**
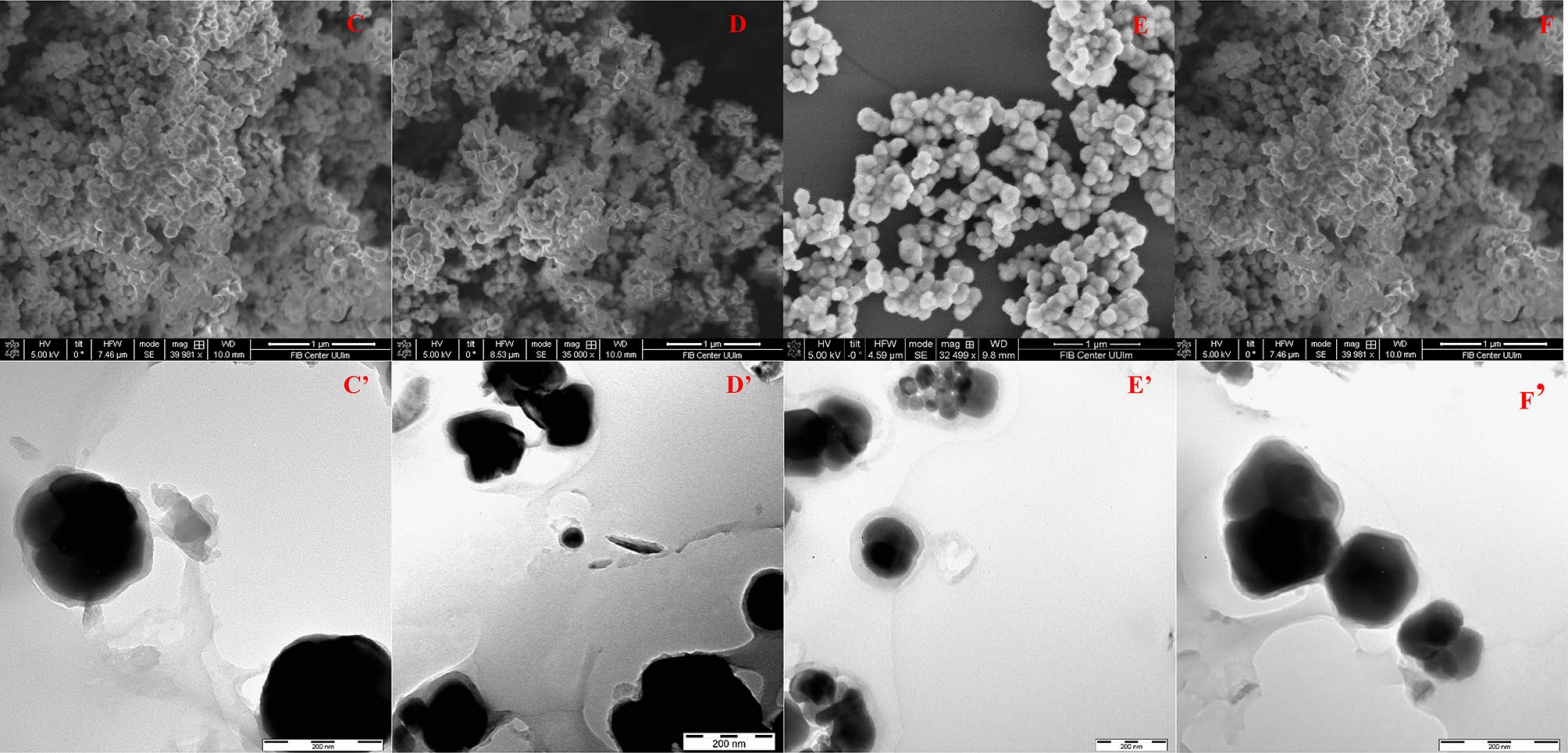
(C) Scanning electron microscopy (SEM) image of MMIP10, (C’) TEM image of MMIP10, (D) SEM image of MNIP10, (D’) TEM image of MNIP10, (E) SEM image of MMIP11, (E’) TEM image of MMIP11, (F) SEM image of MNIP11, and (F’) TEM image of MNIP11, illustrating the morphology and core–shell structure of the polymers.

The morphologies of MMIP10 and MMIP11, along with their corresponding MNIPs, were examined by SEM and TEM. SEM images (Fig. 3C–F) revealed that all samples exhibited predominantly spherical particles with uniform morphology and smooth surfaces, without the appearance of bulky aggregates. Some degree of particle clustering was observed, which can be attributed to magnetic interactions between the particles, in agreement with previous reports on Fe₃O₄–silica composites^69^. TEM analysis further confirmed the presence of polymer films, with average thicknesses of 37.9 ± 13.24 nm for MMIP10 (Fig. 3C’) and 13.8 ± 4.39 nm for MMIP11 (Fig. 3E’). As shown in Fig. 3C’ and D’ a clear increase in thickness relative to the immobilized Fe₃O₄@SiO₂–NH₂@GA@APBA (8.10 ± 3.39 nm) (Fig. 2B) was observed for MMIP10 and its corresponding MNIP, providing evidence for substantial polymer growth; in contrast as shown in Fig. 3E’ and F’ respectively, MMIP11 and MNIP11 showed only modest increases under milder conditions. The increase in thickness observed here is consistent with previous reports on GA-modified APBA-based imprinted systems, where stepwise shell growth has been demonstrated as evidence of successful polymerization^40^.

**Fig. 4:**
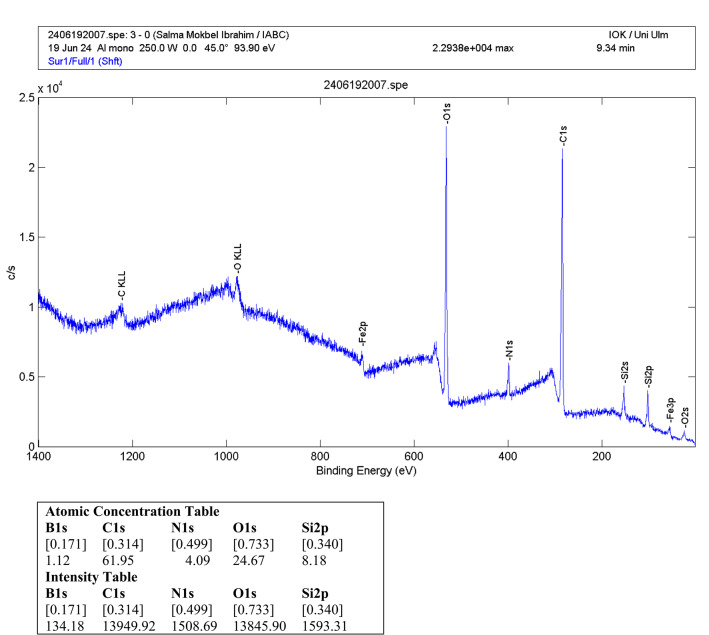
X-ray photoelectron spectroscopy (XPS) survey spectrum of Fe₃O₄@SiO₂–NH₂@GA@APBA, showing characteristic peaks corresponding to B, C, N, O, Si, and Fe, which confirm the successful surface functionalization of the silica-coated magnetite core. The appearance of a distinct boron (B 1 s) signal indicates effective modification of the support. The atomic concentrations obtained from the survey spectrum are listed in the accompanying table.

**Fig. 5:**
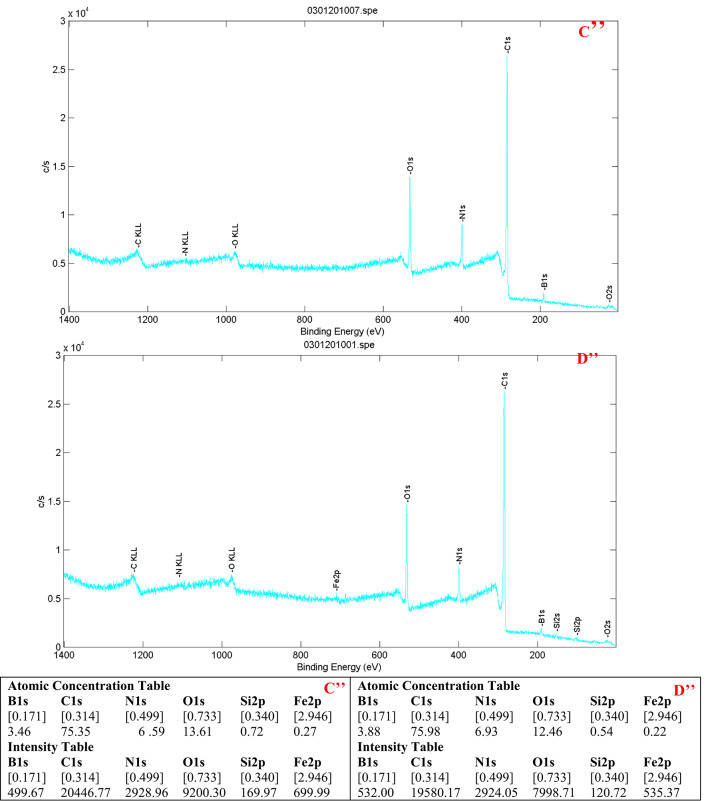
(C’’) XPS survey spectrum of MMIP10 and (D’’) that of MNIP10, showing characteristic peaks of B, C, N, O, Si, and Fe. The increase in the boron (B) signal intensity and atomic percentage relative to Fe₃O₄@SiO₂–NH₂@GA@APBA confirms the successful incorporation of the boronic acid functional monomer into the polymer matrix and the expected surface composition of the imprinted and non-imprinted polymers. The atomic concentrations and intensity values derived from the survey spectra are summarized in the tables below each spectrum.

**Fig. 6:**
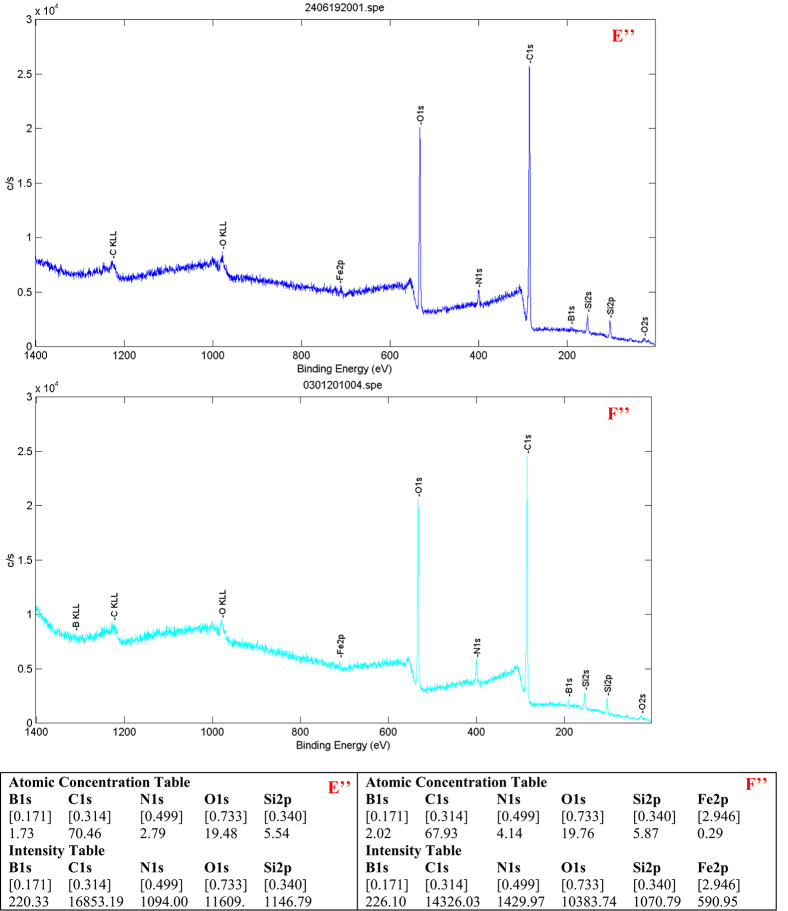
(E’’) XPS survey spectrum of MMIP11 and (F’’) that of MNIP11, showing characteristic peaks of B, C, N, O, Si, and Fe. The increase in boron (B 1 s) signal intensity and atomic percentage relative to Fe₃O₄@SiO₂–NH₂@GA@APBA confirms successful incorporation of the boronic-acid functional monomer and the expected surface composition of the imprinted and non-imprinted polymers. The atomic concentrations and intensity values obtained from the spectra are summarized in the tables below each spectrum.

XPS analysis further confirmed successful polymerization. The boron content rose from 1.12% in Fe₃O₄@SiO₂–NH₂@GA@APBA (Fig. 4) to 3.46% in MMIP10 and 1.73% in MMIP11 (Figs. 5C’’ and 6E’’ respectively). A clear B 1 s (~ 191 eV) signal was consistently observed. This increase indicates the incorporation of APBA units into the polymer layer. Similar use of B 1 s as an indicator of successful polymerization has been reported in APBA-based imprinting systems. These findings provide strong evidence for the formation of polymer layers in both MMIPs and their respective MNIPs. Additionally, MMIP10 and MNIP10 (Fig. 5C’’ and D’’ respectively) exhibited substantially higher boron signals compared to MMIP11 and MNIP11(Fig. 6E’’ and F’’ respectively), again consistent with the more rigorous polymerization conditions used for MMIP10 BET analysis showed comparable surface areas for MMIP10 and MNIP10 (18.86 and 21.74 m² g⁻¹ respectively). BJH pore size analysis gave almost identical diameters (1.991 nm for MMIP10, 1.996 nm for MNIP10). The average pore volumes were also similar: 0.063 cm³ g⁻¹ for MMIPs and 0.078 cm³ g⁻¹ for MNIPs. These findings provide evidence that the higher binding capacity of.

MMIPs arises mainly from the imprinting effect, rather than from surface area or porosity. Similar results have been reported for biochanin imprinted polymers, where MIPs and NIPs showed comparable pore diameters and surface areas, yet the MIPs exhibited markedly higher adsorption due to the imprinting effect^70^.

### Binding isotherms

MMIP10 and its corresponding MNIP10 were incubated with a series of DEX solutions at concentrations ranging from 5.0 × 10^⁻⁶^ to 7.5 × 10^⁻⁴^ M in carbonate buffer (pH 10.0), prepared using UPW. The experimental protocol followed the same procedure described previously. The concentration of unbound DEX in the supernatant was then quantified. The binding isotherms are presented in Figure S7A. They illustrate the relationship between the amount of DEX bound to the polymers and the initial DEX concentration. As expected, the amount of DEX bound increased with concentration, reaching saturation at approximately 5.0 × 10^⁻⁴^ M. The difference in binding performance between MMIP10 and MNIP10 was most pronounced within the range of 2.5 × 10^⁻⁵^ to 2.5 × 10^⁻⁴^ M. This provides evidence for the contribution of imprinted cavities to specific binding. At concentrations above 2.5 × 10^⁻⁴^ M, the binding behavior of the two polymers became more comparable, likely due to site saturation.

To further study the adsorption mechanism, the MMIP10 data were analyzed using both Langmuir and Freundlich models^71^. The Langmuir model assumes monolayer adsorption on a surface with homogeneous sites. In contrast, the Freundlich model describes adsorption on heterogeneous surfaces and multilayer adsorption.¹ The MMIP10 data fitted the Langmuir model better, with R^²^ = 0.9698 compared to R^²^ = 0.9384 for Freundlich. This is also evident from the fitted curves. The Langmuir model is shown in Figure S8 and the Freundlich model in Figure S9. From the Langmuir model, q_max_ was 500.0 µmol g^⁻¹^ and KL was 6.11 × 10^⁻⁴^ L·µM^⁻¹^. The relatively high q_max_ reflects the abundant density of imprinted binding sites in MMIP10. This agrees with reports showing that high q_max_ values are linked to sensitive MIPs^72^. According to the Freundlich model, KF was 2.2 and 1/n was 0.8531. The 1/n value, close to 1, indicates nearly linear adsorption. This supports the presence of relatively homogeneous recognition sites, consistent with the interpretation of Freundlich constants in the literature^73^. Together, these results highlight the high capacity, strong affinity, and homogeneous binding environment of MMIP10.

### Binding kinetics

The rate at which DEX is adsorbed and equilibrium binding is achieved is a critical parameter in evaluating MMIPs, as it reflects the time required to reach maximum adsorption of the template molecules. As illustrated in Figure S7B, MMIP10 exhibited rapid adsorption kinetics, achieving 14.66% binding within just 7 min. In contrast, the corresponding MNIP showed only 3.66% binding during the same time interval. Providing evidence for the presence of specific binding sites and the occurrence of selective interactions in MMIP10.

This rapid initial binding observed for MMIP10 is likely due to the easy accessibility of recognition sites, characteristic of surface imprinting techniques. The greater binding efficiency of MMIP10 compared to MNIP10 in the early stages further supports the interpretation that specific binding occurs more rapidly than nonspecific interactions.

Equilibrium binding was achieved within 2 h for MMIP10, with a maximum binding percentage of 67.02%. In contrast, MNIP10 had not reached equilibrium within the same period, indicating the presence of high-affinity, selective binding sites in MMIP10 that contribute to faster and more efficient adsorption. This fast mass transfer and high binding efficiency suggest that MMIP10 holds strong potential for practical applications, such as SPE and chromatographic separations^74^.

To further elucidate the adsorption mechanism, the kinetic data were analyzed using the PFO model of Lagergren (1898) and the PSO model of Ho and McKay (1999). The corresponding regression plots and correlation coefficients (R²) are provided in Supplementary Figures S10 and S11 respectively. The PFO model provided the best fit, with a high correlation coefficient (R² = 0.9987) and a calculated adsorption capacity (q_e_,calc = 10.81 µmol g⁻¹) that was in close agreement with the experimental value (q_e_,exp = 11.01 µmol g⁻¹). In contrast, the PSO model gave a lower correlation (R² = 0.9902) and overestimated q_e_,calc at 13.96 µmol g⁻¹. These results provides evidence that the adsorption of DEX onto MMIP10 follows PFO, indicating that uptake is dominated by rapid occupation of surface-imprinted recognition sites^48,49^.

Similar results were reported by Yao et al. (2024). Their carbon nanotubes based MMIPs for glucose also fit the PFO model (R² ≈ 0.942), with equilibrium reached in ~ 50–70 min. The smaller glucose size and dual-site binding likely accounts for the faster uptake compared to DEX^20^.

Ali et al. (2024) observed even shorter equilibrium times (~ 25–30 min) for malathion and chlorpyrifos using fluorescent magnetic MMIPs. Both pesticides followed PFO kinetics with high correlations (R² = 0.992–0.998)^75^. The small molecular size and porous polymer structure supported rapid diffusion.

Across these systems, surface imprinting consistently favors PFO kinetics. The differences in equilibrium time reflect analyte size, polymer porosity, and site heterogeneity. The strong PFO fit in our work indicates efficient surface imprinting and highlights the potential of MMIP10 for rapid and selective adsorption^20,75^.

### Selectivity studies

**Fig. 7:**
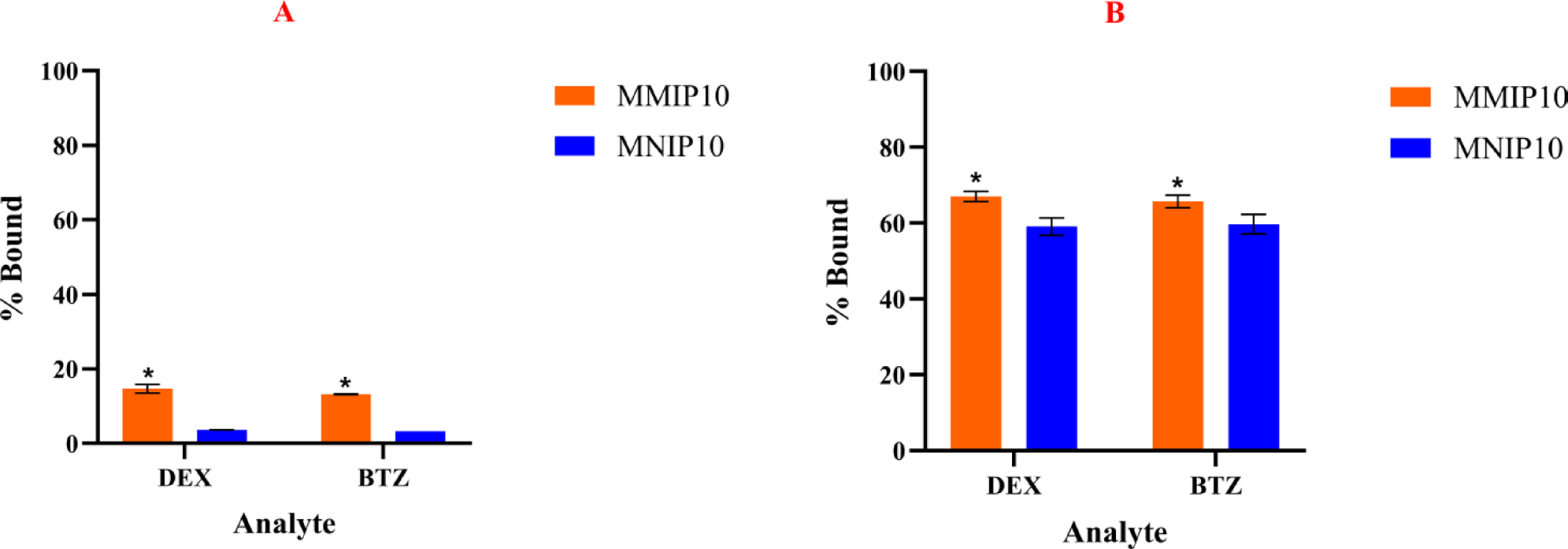
(**A**) Percentage of DEX and betamethasone (BTZ) bound to polymer 10 after 7 min of incubation, expressed relative to the initial analyte concentration of 5.0 × 10⁻⁵ M. (**B**) Percentage of DEX and BTZ bound to polymer 10 after 2 h of incubation, expressed relative to the same initial analyte concentration. In each experiment, 10 mg of sorbent was incubated with 3 mL of analyte solution. All experiments were performed in triplicate (*n* = 3), and error bars represent ± SD. * Indicates a statistically significant difference between each MMIP and its corresponding MNIP (unpaired two-tailed t-test, *p* < 0.05).

A noncompetitive binding study was carried out to assess the ability of MMIP10 to differentiate between the two isomers, DEX and BTZ. The binding percentages of both compounds were nearly identical at 7 min and 2 h as shown in Fig. 7A and B respectively. Statistical analysis confirmed no significant difference between DEX and BTZ binding (*p* > 0.05). These results indicates that MMIP10 lacks chiral selectivity.

The polymer still showed good binding toward both isomers, which suggests its potential for extraction of these compounds. The selectivity coefficients (α) were 1.11 at 7 min and 1.02 at 2 h, while the IFs were 0.99 and 1.02. These values confirm the absence of stereoselectivity. Reproducibility was high, with RSD < 5%.

### UPLC–MS/MS method development

A new UPLC–MS/MS method was developed to quantify DEX. DEX had previously been reported as an internal standard for the determination of TCA, so TCA was selected as an internal standard in this method^80^.

Notably, for the quantitative LC‒MS method, DEX is not highly ionizable; however, the response in positive mode was better than that in negative mode, and was therefore selected. This may be because both DEX and TCA contain a carbonyl group that can be protonated, resulting in a mass response. The precursor ion [M + H] ^+^, where M is the molecular mass of the respective analyte, is formed by the addition of a proton to generate the positively charged molecular ion. The parent ions were detected in the mass spectra at m/z 393.1 for DEX and m/z 435.1 for TCA. The capillary voltage was optimized, and 3.06 kV was selected for the analysis of both compounds, as it resulted in the highest mass response of the parent ions. A gradual increase in the collision energy was used to optimize the formation of daughter ions.

**Fig. 8:**
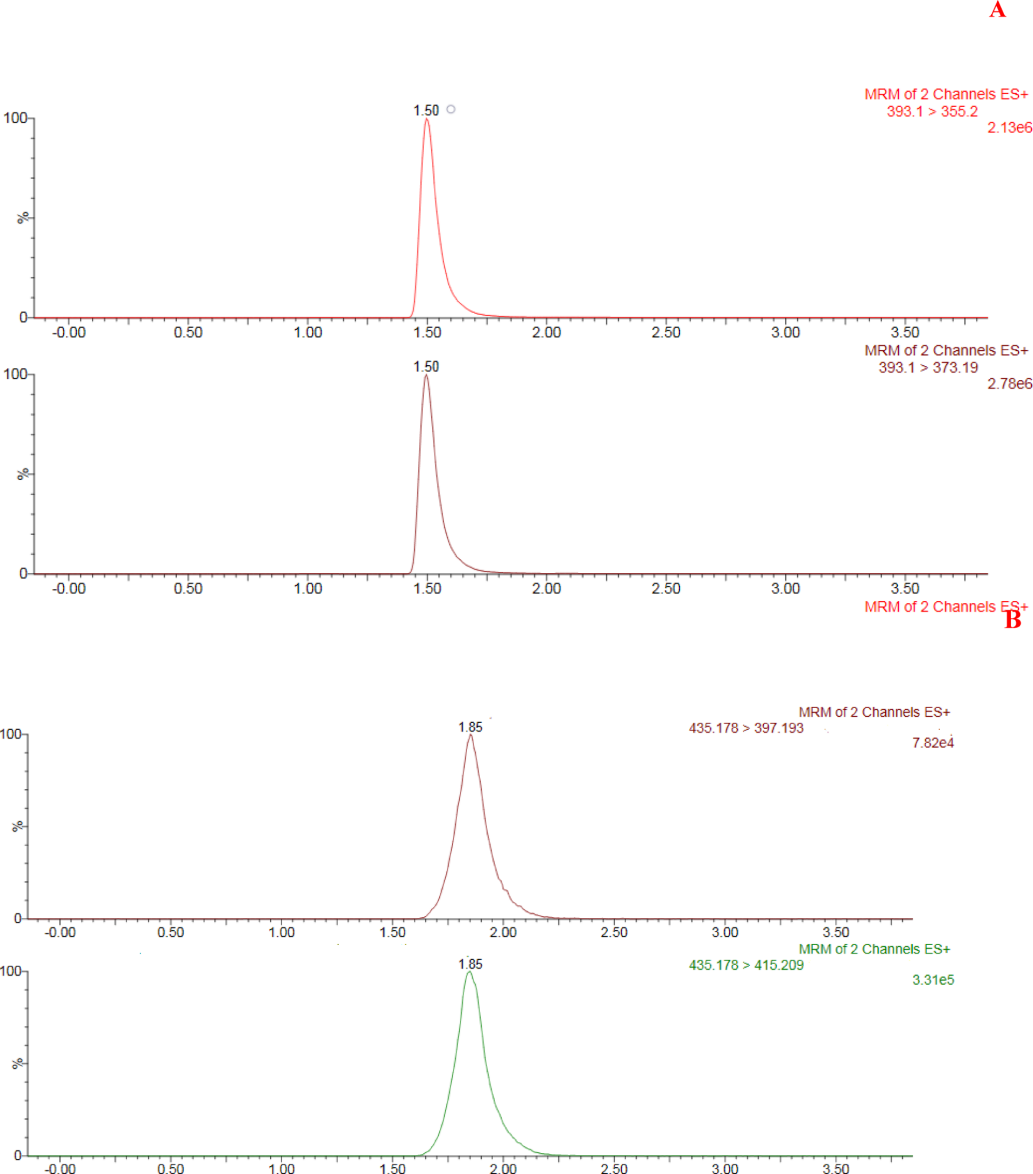
(**A**) Chromatogram of 2.5 × 10⁻⁶ M DEX acquired in two multiple reaction monitoring (MRM) channels. (**B**) Chromatogram of 2.3 × 10⁻⁶ M triamcinolone acetonide (TCA), used as the internal standard, acquired in two MRM channels under the same analytical conditions.

As shown in Fig. 8A two DEX daughter ions were detected at m/z 373.19 and 355.2 with high sensitivity in the MRM. Higher sensitivity was achieved by using an MRM of 393.1 > 373.19 at a collision energy of 20 V; accordingly, it was selected for use in the quantification. For the internal standard TCA, the daughter ion peak of the highest ion count was found at m/z 415.209, as shown in Fig. 8B; hence, MRM 435.178 > 415.209 (collision energy of 30 V) was selected for quantification.

### UPLC–MS/MS method validation

**Fig. 9:**
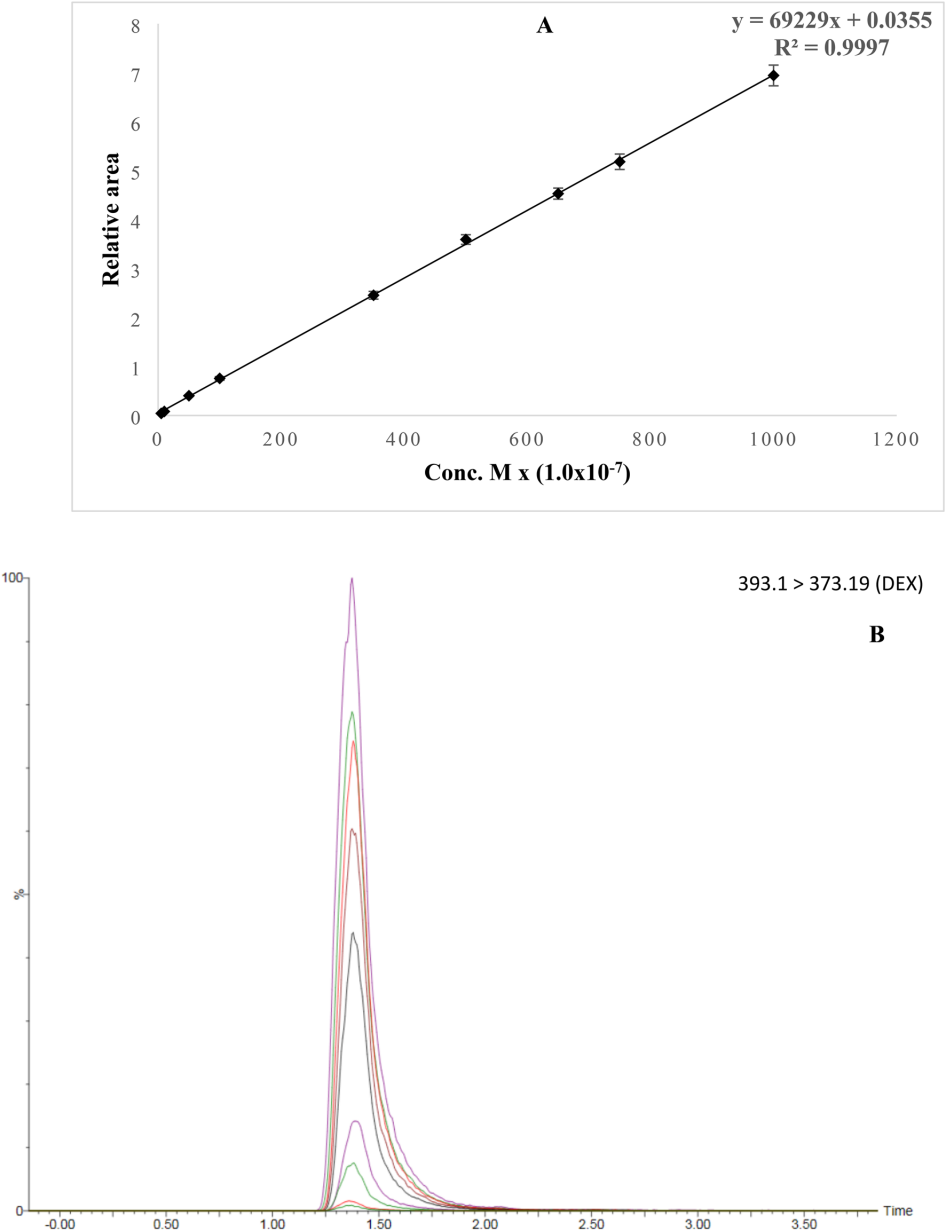
(**A**) Calibration curve of DEX constructed over the concentration range of 5.0 × 10⁻⁷ to 1.0 × 10⁻⁴ M using UPLC–MS/MS analysis. (**B**) Representative chromatograms corresponding to the same DEX concentration range at m/z 373.19, showing the daughter-ion signal.

The newly developed UPLC–MS/MS method was validated in accordance with ICH guidelines, assessing linearity, LOD, LOQ, precision (intra- and interday), and accuracy. As illustrated in Fig. 9A, the method exhibited excellent linearity over the concentration range of 5.0 × 10⁻⁷ to 1.0 × 10⁻⁴ M, with RSD values of 6.28% and 7.28% for the slope and intercept, respectively. The calibration curve followed the equation *y* = 69229*x* + 0.0355, with a R² = 0.9997. Representative chromatograms corresponding to this concentration range for DEX, monitored at m/z 373.19 (daughter ion), are shown in Fig. 9B.

The precision of the method was assessed based on the RSD of both peak area and retention time (RT). For intraday precision, the RSD of the peak area ratio ranged from 0.04% to 3.37%, while the RSD of the.

RT ranged from 0.35% to 1.29%. For interday precision, the RSD of the peak area ratio ranged from 0.61% to 3.27%, and for the RT, from 0.96% to 3.95%.

Accuracy was evaluated by analyzing four concentrations of DEX (1.5 × 10⁻⁵, 5.0 × 10⁻⁵, 6.5 × 10⁻⁵, and 7.5 × 10⁻⁵ M), and recovery rates ranged from 93.39% to 111.68%, with RSD values between 0.02% and 3.43%. The method demonstrated high sensitivity, with an LOD of 1.11 × 10⁻⁷ M and an LOQ of 3.72 × 10⁻⁷ M.

### MISPE of DEX from nile river water sample

Three mL of 5 × 10⁻⁵ M DEX solution, equivalent to 58.86 µg of DEX spiked into Nile River water, was incubated with 20 mg of MMIP10 or MNIP10.

sorbent for 2 h. Following incubation, the particles were washed for 30 min using UPW. Water was selected as the washing solvent to remove interfering substances that might be nonspecifically adsorbed onto the polymer without significantly eluting DEX, given MMIP10’s relatively high affinity for the template.

molecule. In contrast, MNIP10, with its lower affinity, was expected to retain DEX mainly through nonspecific interactions, making it more susceptible to removal during washing.

**Fig. 10:**
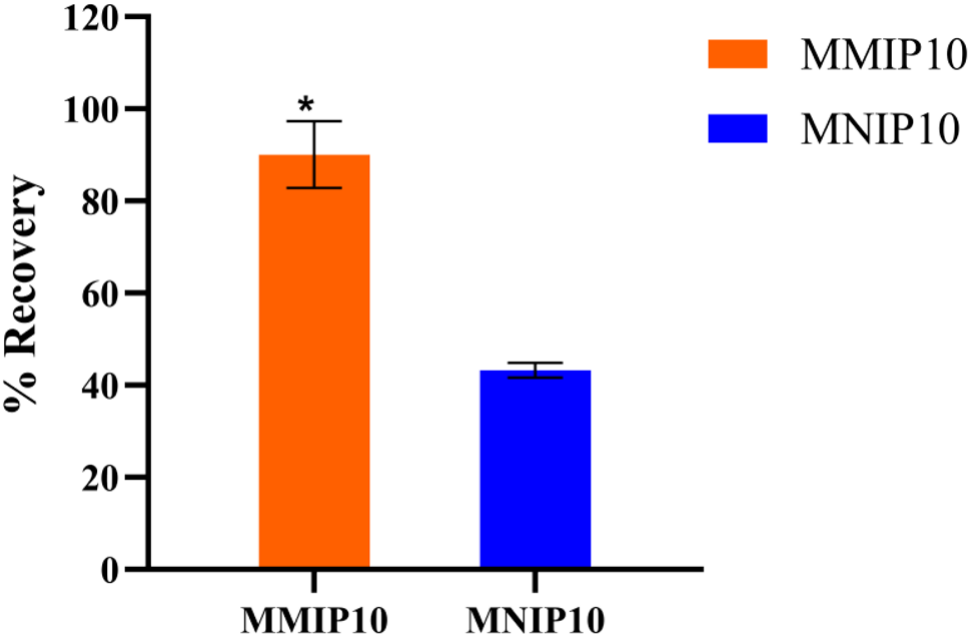
Recovery (%) of DEX from Nile River water spiked at an initial analyte concentration of 5.0 × 10⁻⁵ M using polymer 10 as the sorbent in solid-phase extraction (SPE). For each experiment, 3 mL of the spiked sample was incubated with 20 mg of MMIP10 or MNIP10 for 2 h. * Indicates a statistically significant difference between each MIP and its corresponding NIP (unpaired two-tailed t-test, *p* < 0.05). All experiments were performed in triplicate (*n* = 3), and error bars represent ± SD.

To recover the bound DEX, an elution step was conducted using varying concentrations of AcOH in methanol (5%, 10%, 15%, and 20%). Recovery increased with AcOH concentration, reaching a maximum at 15%. Accordingly, 15% AcOH in methanol was selected as the optimal elution solvent and applied for 2 h. Under these conditions, DEX recovery from MMIP10 was 90.08% ± 7.25 (RSD = 8.05%), whereas recovery from MNIP10 was significantly lower at 43.23% ± 1.59 (RSD = 3.68%). The calculated IF was 2.0, as illustrated in Fig. 10; Table 2.

**Table 2:** Recovery (%) of DEX from tap water spiked at an initial analyte concentration of 5.0 × 10⁻⁵ M using polymer 10 as the sorbent in solid-phase extraction (SPE).

Polymer	B (µmol g^−1^)	Recovery (%)	RSD (%)	IF
MMIP10	6.76	90.08 ± 7.25	8.05	2.2
MNIP10	3.24	43.23 ± 1.59	3.68	

*Values are mean ± SD, *n* = 3. IF = imprinting factor.

Notably, there was no significant difference in the amount of DEX bound after the initial incubation step, which further highlights the critical role of the washing step in removing nonspecifically bound drug molecules.

These findings provide strong evidence of the successful imprinting of MMIP10 and the presence of selective recognition sites within the polymer matrix.

### Greenness of synthesis and SPE workflow

To evaluate the environmental footprint of the MISPE procedure, both SI and AMVI were calculated. For the MMIP workflow, SI was 3.5 mL organic solvent per sample (3.0 mL MeOH/AcOH for elution and 0.5 mL ACN for reconstitution), while AMVI was 9 mL (3.5 mL organic plus 5.5 mL aqueous). By contrast, activated-carbon SPE protocols typically consume 6–15 mL organic solvent, and their AMVI increases to 16–25 mL once aqueous washing and conditioning are included^76^. Reversed-phase C18 cartridges or disks require about 5–15 mL organic solvent (SI), with a corresponding AMVI of 15–25 mL^77,78^. In a DEX-specific method using Sep-Pak Plus C18 cartridges (500 mg), at least 5 mL organic solvent was used for elution alone. Including aqueous steps increases AMVI to ≥ 15 mL^79^. Taken together, these comparisons demonstrate that the MMIP-based MISPE protocol achieves substantially lower SI and AMVI values than conventional sorbents, supporting its greener profile for DEX extraction.

Although reusability was not evaluated in the present study, the reusability of MMIPs is well documented. For example, Meseguer-Lloret et al. (2022) reported that magnetic MIP sorbents could be reused for up to 65 cycles while maintaining excellent selectivity^80^. This feature not only reduces overall resource consumption but also minimizes solid waste generation compared with one-time-use sorbents such as activated carbon or C18 cartridges. In addition, regeneration of MMIPs relies on moderate solvent volumes rather than the harsh acid or chlorinated treatments often employed for other adsorbents, thereby lowering the burden of hazardous waste disposal.

The greenness of the MMIP10 synthesis was further assessed using the EcoScale evaluation method. This tool assigns a score out of 100 by deducting penalty points for low yield, hazardous reagents, demanding conditions, or extensive work-up. Scores of ≥ 75 are classified as excellent, 50–74 as acceptable, and < 50 as inadequate. The three synthesis steps (amination, GA/APBA immobilization, and polymerization) scored 89, 90, and 89, respectively. Giving an overall value of ~ 89 that places the route firmly in the excellent category. Deductions were mainly linked to the use of ammonia solution, GA, sodium borohydride, ACN, methanol, and AcOH, as well as to heating during polymerization^53,54^.

The SPE step was evaluated in terms of greenness using the AGREEprep and Eco-Scale tools. AGREEprep was used to compare the SPE step of three workflows that use different sorbent types: a magnetic MIP sorbent (used in this work), a conventional silica-based C18 sorbent^55^, and a Polymeric HLB sorbent (Oasis HLB)^56^. As shown in Figure S13 the MMIP-based method scored 0.51, which lies in the moderate greenness range. The Oasis HLB-based method scored 0.33 and the C18-based method scored 0.14, both of which are classified as poor. Eco-Scale was also applied to the same three SPE workflows. As shown in Table S1 the MMIP-based method achieved a score of 77 (excellent), whereas the Oasis HLB and C18 methods scored 62 and 52, respectively, which fall in the acceptable range.

This superiority is attributed to the lower solvent consumption, reduced waste, and the minimization of hazardous solvents in the MMIP protocol. In the AGREEprep assessment, the MMIP sorbent was reported as non-reusable for transparency, although MMIPs are typically reusable, which means the current score is conservative and likely underestimated. In addition, the use of UPLC–MS/MS in this study contributed to a lower post-preparation score, even though the workflow is not inherently dependent on UPLC–MS/MS and could be combined with less resource-intensive detectors. Therefore, the actual greenness advantage of the MMIP-based SPE procedure is expected to be even greater than the scores indicate.

### Comparison with reported MIPs

After comparing the extraction efficiency of the MMIP with conventional sorbents, the present work was further compared with previously reported DEX-selective MIPs to evaluate the entire process rather than isolated steps. This comparison was necessary to determine whether replacing the conventional monomer and organic media with a water-based system can still deliver a practical, effective, and greener SPE method for DEX. Earlier DEX MIPs were largely built on methacrylic acid and organic solvent systems. More recent variants introduced deep eutectic-solvent–derived magnetic MIPs^34^. and a non-magnetic hydrogel for water matrices based on 2-hydroxyethyl methacrylate^36^. In contrast, the APBA-based magnetic MMIP10 was designed around water, employing it both as the porogen during synthesis and as the binding solvent, while still maintaining high recovery. The solvent selection guide (Figure S14) reinforces this choice: water is rated “recommended,” whereas methanol, ACN, and AcOH used previously are flagged as problematic or hazardous.

Table 1 also highlights the kinetic behavior of the compared systems. For previously reported MIPs, the onset of adsorption typically appeared within a few minutes (~ 5 min), and a similar early uptake was observed for MMIP10 after ~ 7 min. Equilibrium for MMIP10 was reached within 120 min, which is shorter than or comparable to reported intervals (120–180 min) This confirms that switching to a water-centered design does not impose a kinetic penalty and is consistent with the intrinsically fast binding kinetics typically reported for MIPs.

To enable a holistic comparison that accounts for both performance and sustainability, the AGREEmip metric was calculated for each system individually. As shown in Figure S15 the reported scores were 0.50 (Liu et al., 2017), 0.67 (Du et al., 2018), 0.62 (Hang et al., 2022), 0.61 (Adauto et al., 2025), and 0.63 for MMIP10 in this study. Within this scale, scores closer to 1 reflect stronger adherence to green-chemistry criteria embedded in MIP design, whereas values near 0 indicate poor alignment. The score of MMIP10 (0.63) therefore falls within the same greenness class as the highest-scoring literature protocol while being constructed under fully water-based conditions.

Although reusability was not experimentally examined in this study, the polymer was intentionally reported as single-use for transparency. This does not imply a structural limitation, as MMIPs are widely documented to retain performance over multiple adsorption–desorption cycles in comparable systems, as seen in the literature in Table 1. Likewise, ACN was used solely for dissolving the drug template, which exhibits limited solubility in water, and its presence is not essential for the polymerization step itself. These two factors would shift MMIP10 into a more favorable category if reuse were validated and solvent substitution implemented.

### Limitations

Although this study clearly demonstrates that MMIPs can remove DEX with high efficiency and rapid magnetic separation, several limitations should be acknowledged. The experiments were mainly conducted in tap water, which is less complex than real environmental matrices such as wastewater or river water that contain higher organic loads and multiple co-existing pharmaceuticals. In addition, the study was performed at relatively high DEX concentrations, and further evaluation at environmentally relevant trace levels is needed. Theoretically, MIPs are characterized by their reusability; however, in this work, the regeneration potential of the MMIPs was not fully examined. It also remains to be clarified whether the developed material can effectively capture other structurally related glucocorticoids. Addressing these aspects in future studies will provide a more comprehensive understanding of the applicability and robustness of the proposed approach and how versatile this method can be for real-world applications.

## Conclusions

In this study, MMIPs are synthesized using APBA and evaluated for their rebinding ability. Adsorption isotherms and binding kinetics are established, and the polymer is applied for the extraction of DEX from Nile River water, achieving recoveries above 90% (± 7.25%). A comparison with the corresponding NIP shows an IF of 2.21, providing evidence of the specificity of the polymer.

Quantification of DEX is performed using a newly developed UPLC–MS/MS method validated according to ICH guidelines. The method shows a green profile (SI = 3.5 mL; AMVI = 9.0 mL), with excellent synthesis greenness (EcoScale ≈ 89; AGREEMIP = 0.63) and favorable MSPE performance (EcoScale = 77; AGREEprep = 0.51). Compared with previously reported methods, the current approach provides higher recovery, aligns with green chemistry principles, and operates under an external magnetic force without requiring time-consuming procedures.

## Supplementary Information

Below is the link to the electronic supplementary material.

Supplementary Material 1

## Data Availability

The data supporting the findings of this research are available within the article and its supplementary information.
