# Supplementary material for: Development of a magnetic molecularly imprinted polymer for the removal of dexamethasone from river nile water
Source: Sci Rep. 2025 Dec 29;15:45661. doi: 10.1038/s41598-025-32056-0 (PMC12753826; doi:10.1038/s41598-025-32056-0)
Supplement: Supplementary file 1 — Supplementary Material 1 [file 41598_2025_32056_MOESM1_ESM.docx]

**Development of a magnetic molecularly imprinted polymer for the removal of dexamethasone from River Nile water**

Salma A. Mokbel^1,2^, [Mohammad Abdel-Halim](https://sciprofiles.com/profile/1194383)^2^, Mehmet Dinc^3^, Boris Mizaikoff^3.4^ and [Nesrine A. El Gohary](https://sciprofiles.com/profile/1151209)^2^


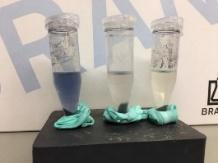


**Figure S1.** Ninhydrin color test confirming the stepwise surface modification of the magnetic core. The **violet color** of **Fe₃O₄@SiO₂–NH₂** (left) indicates **successful amination** and the presence of **free surface amine groups**, while both **Fe₃O₄@SiO₂–NH₂@GA** (middle) and **Fe₃O₄@SiO₂–NH₂@GA@APBA** (right) appear transparent, consistent with the **absence of accessible amine functionalities** following subsequent **immobilization steps**

**Figure S2.** Schematic representation of the synthetic pathway of MMIP10, illustrating the surface coating of Fe₃O₄ nanoparticles with silica and amino functionalization using TEOS and APTES, followed by surface coupling with glutaraldehyde and immobilization of 3-aminophenylboronic acid (APBA) via imine bond formation. The final reduction step with NaBH₄ stabilizes the linkage, yielding the APBA-functionalized magnetic core (MMIP10 precursor).

**
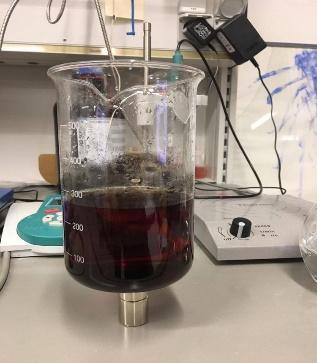

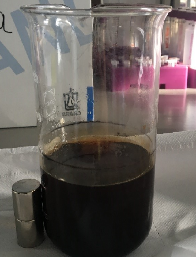
**

**Figure S3.** Polymerization media of **MMIP11** (left) and **MMIP10** (right) after completion of the polymerization process, showing a noticeable difference in **color intensity**, with **MMIP10 exhibiting a darker appearance**, suggesting a **higher polymer content** compared to **MMIP11**.

**Figure S4.** Schematic representation of the binding mechanism of phenylboronic acid with diol-containing molecules under **neutral and alkaline pH conditions**.^1^

**
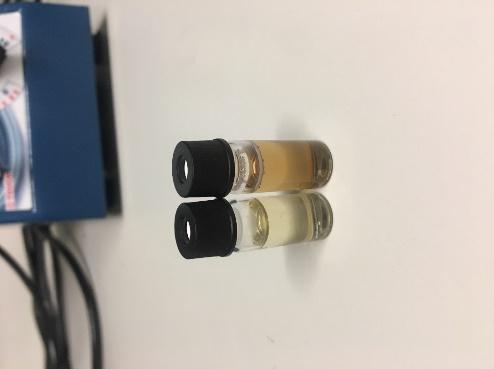
Figure S5.** Supernatants of the binding solutions prepared in ultrapure water (UPW) at pH 10.0 (left) and pH 12.0 (right), respectively. The brown coloration observed at pH 12.0 indicates significant polymer leaching, whereas the solution at pH 10.0 remained nearly clear. Based on these observations, pH 10.0 was selected as the optimal condition for the rebinding experiments.

**Figure S6**. Schematic representation of the proposed adsorption mechanism of DEX on MMIP10, illustrating the stabilization of the DEX–MMIP10 complex through boronate ester formation (orange), hydrogen bonding (blue), and hydrophobic interactions (black arrows).


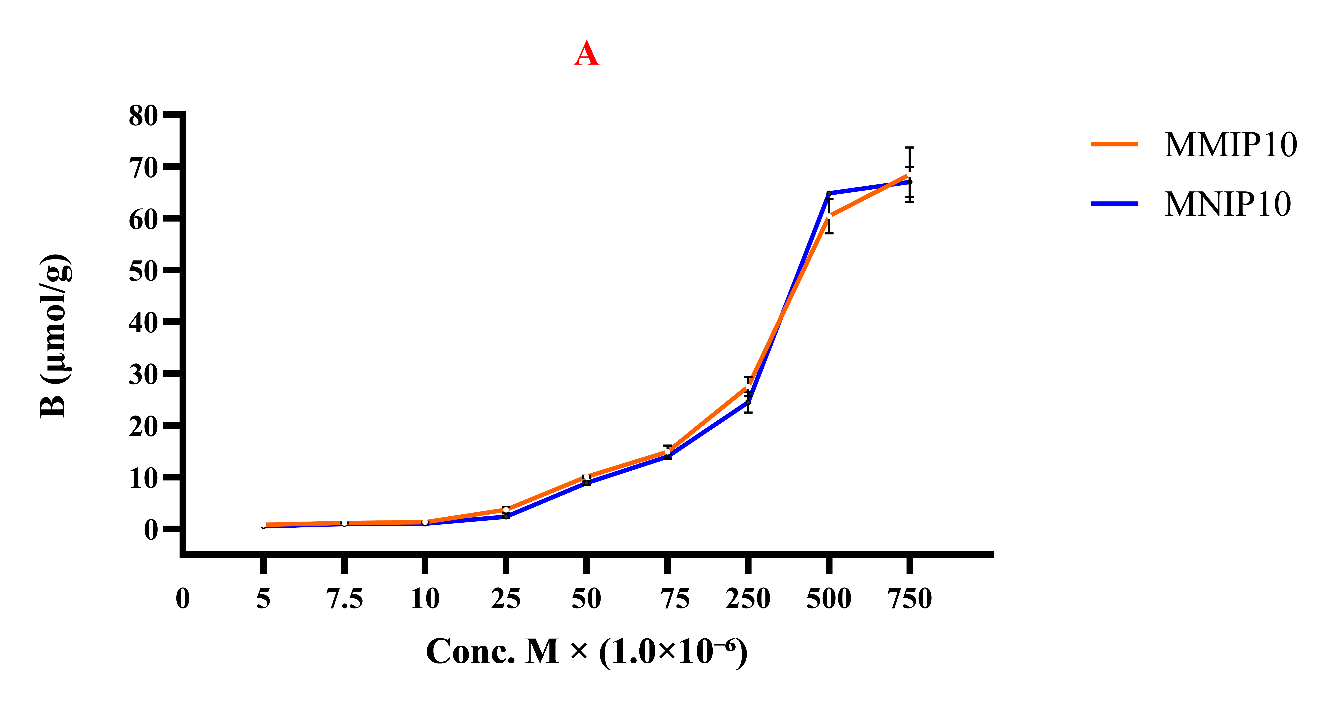


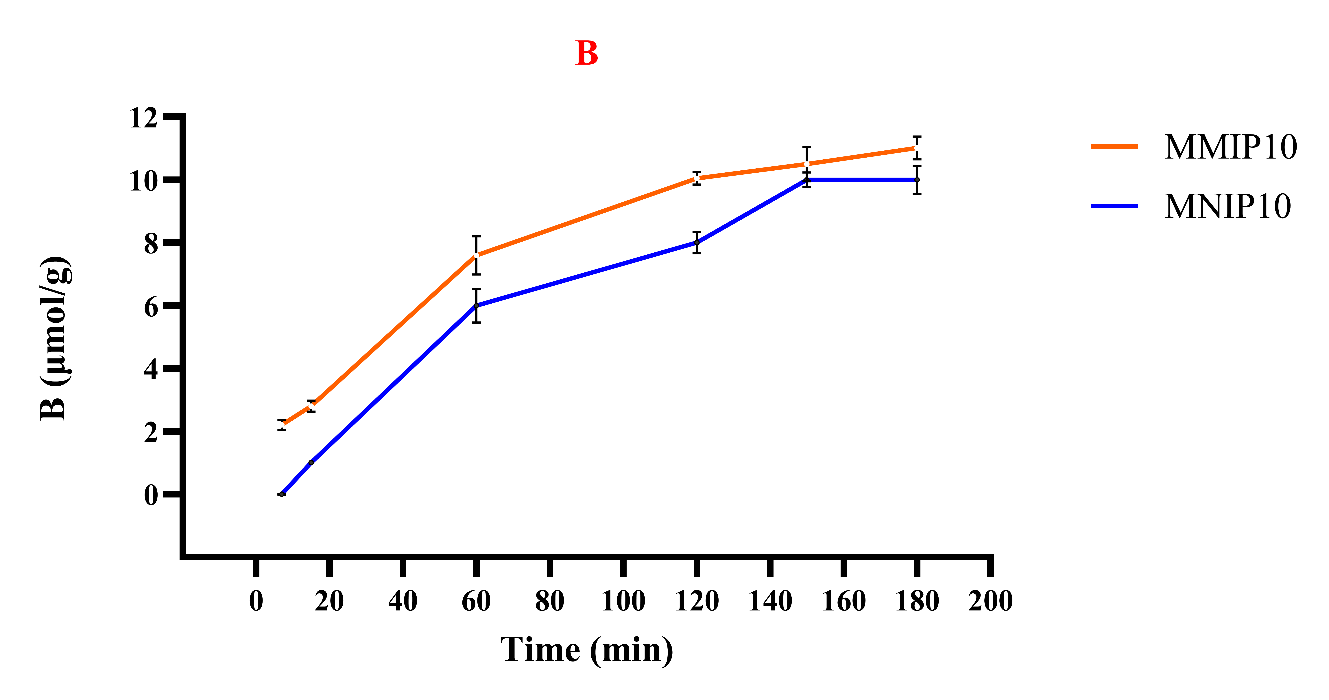


**Figure S7.** (A) Adsorption isotherms of **MMIP10** and **MNIP10** obtained over the concentration range of **5.0 × 10⁻⁶ to 7.5 × 10⁻⁴ M** in **carbonate buffer (pH 10.0)**, using **10 mg of polymer** and **3 mL of DEX solution** with an incubation time of **2 h.** (B) Binding kinetics of **MMIP10** and **MNIP10** measured at a fixed **DEX concentration of 5.0 × 10⁻⁵ M** in **3 mL of carbonate buffer (pH 10.0)** over a time range of **3–180 min**. All experiments were performed in **triplicate (n = 3)**, and error bars represent **± SD**.

**Figure S8.** Langmuir isotherm model fitting for the adsorption of DEX onto MMIP10, showing the correlation between the experimental and theoretical adsorption capacities.

**Figure S9.** Freundlich isotherm model fitting for the adsorption of DEX onto MMIP10, showing the correlation between the experimental and theoretical adsorption capacities.

**Figure S10.** Pseudo-first-order kinetic model fitting for the adsorption of DEX onto MMIP10, showing the experimental adsorption data and the corresponding kinetic model fit.

**Figure S11.** Pseudo-second-order kinetic model fitting for the adsorption of DEX onto MMIP10, showing the experimental adsorption data and the corresponding kinetic model fit.


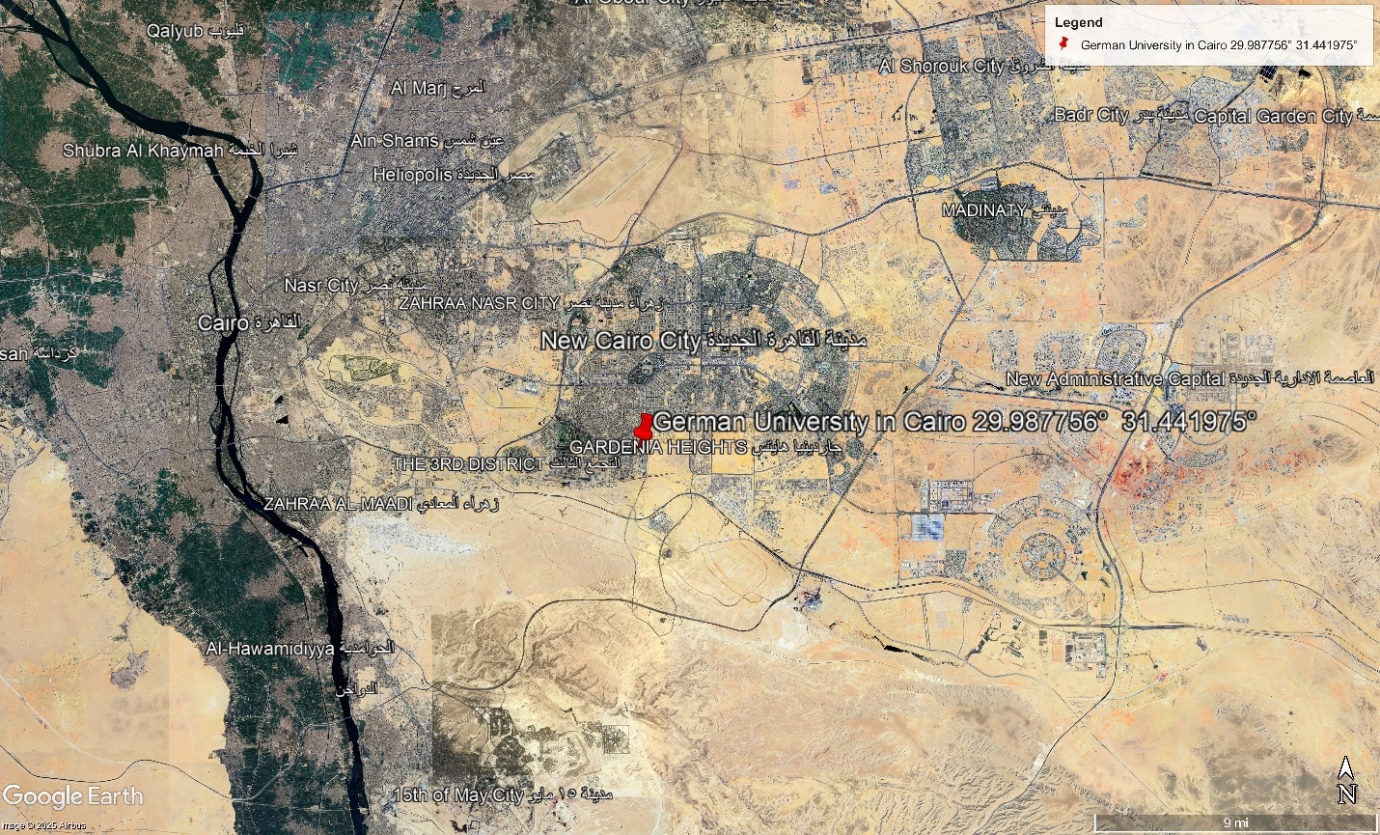


**Figure S12.** Sampling site for tap water collection at the German University in Cairo (GUC), 5th Settlement, New Cairo City, Cairo Governorate, Egypt (**29.987756° N, 31.441975° E**). Map generated using **Google Earth Pro, version 7.3** (© Google; https://www.google.com/earth/versions/#earth-pro; accessed on 26 November 2025).


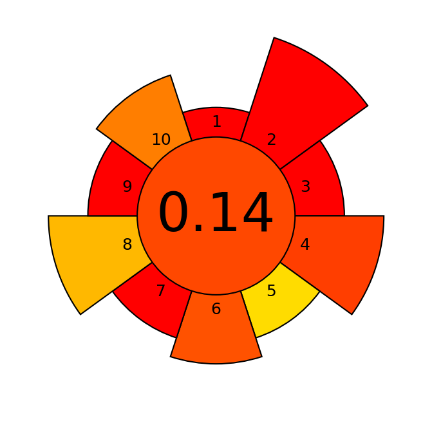

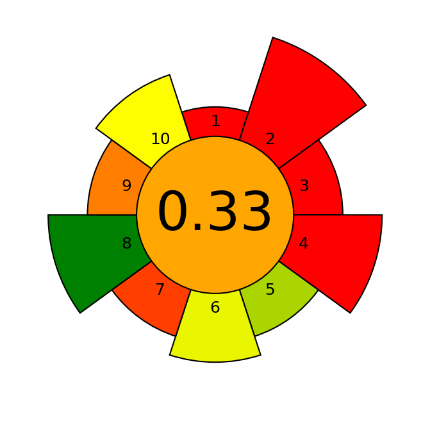

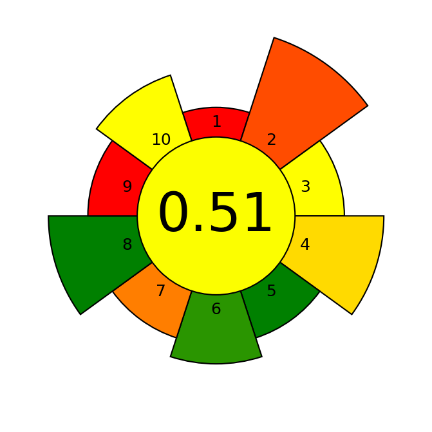
 **Figure S13. AGREEprep evaluation of three SPE workflows using different sorbents.** (A) C18 silica-based sorbent (score = 0.14), (B) Polymeric HLB sorbent (Oasis HLB) (score = 0.33), and (C) Magnetic MIP-based SPE used in this work (score = 0.51). The numbered radial segments represent the AGREEprep criteria: (1) sample-preparation placement, (2) hazardous materials, (3) sustainability/renewability/reusability of materials, (4) waste generated, (5) size economy of the sample, (6) sample throughput, (7) degree of automation/integration, (8) energy consumption, (9) post-preparation configuration, and (10) operator’s safety. Colors reflect the relative greenness of each criterion, with green indicating favorable performance and red indicating unfavorable performance. AGREEprep provides 0–1 score that reflect the extent of alignment with green chemistry principles, with higher values indicating better greenness**.^3^**

**A**

**C**

**B**

| **Table S1**: Eco-Scale evaluation of the three SPE workflows using different sorbents C18 silica-based SPE, HLB polymeric SPE, and (MMIP magnetic SPE). The table lists the penalty points assigned to each category and the resulting Eco-Scale scores used to classify the overall greenness of each workflow. | | | | |
| --- | --- | --- | --- | --- |
| **Category** | **Evaluation criterion** | **C18 silica SPE**^4^ | **Polymeric HLB (Oasis HLB)**^5^ | **MMIP10 magnetic SPE (this work)** |
| Reagents (types) | Number/diversity | Several  Penalty = 6 | Very few Penalty = 0 | Few reagents Penalty = 3 |
| Total solvent volume | Total used in SPE | 23 mL  Penalty = 3 | 60 mL  Penalty = 17 | 8.5 mL  Penalty = 1 |
| Energy use | Drying/heating/vacuum | None  Penalty = 0 | Vacuum Penalty = 1 | None  Penalty = 0 |
| Hazard profile | Toxic/flammable solvents | MeOH + hexane Penalty = 10 | MeOH  Penalty = 8 | MeOH  Penalty = 8 |
| Waste & treatment | Waste amount & handling | Moderate waste Penalty = 2 | High waste Penalty = 7 | Low waste Penalty = 2 |
| Instrumentation | Device / disposables | Reusable cartridge  Penalty = 2 | Disposable cartridge  Penalty = 5 | Magnet; single-use  Penalty = 4 |
| Recovery (%) | Penalty = (100−%) /2 | ~50%  Penalty = 25 | ~100%  Penalty = 0 | ~90%  Penalty = 5 |
| Σ Penalty points |  | 48 | 38 | 23 |
| Eco-Scale (100−Σ) |  | 52  Acceptable | 62  Acceptable | 77  Excellent |


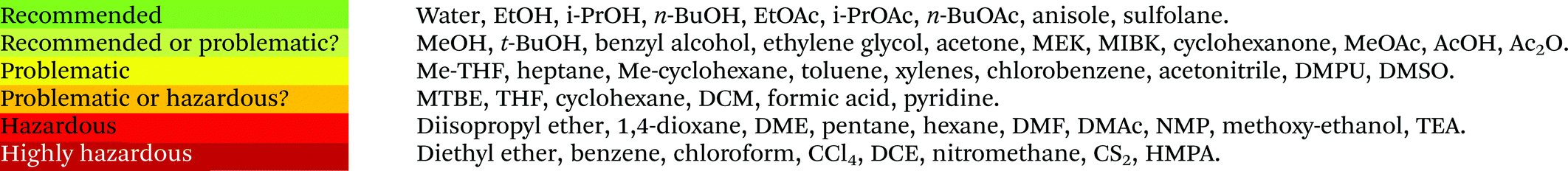


**Figure S14**. CHEM21 solvent selection guide. Solvents are categorized according to their environmental, health, and safety profiles as follows: recommended (green), recommended/problematic (light green), problematic (yellow), problematic/hazardous (orange), hazardous (red), and highly hazardous (dark red).^2^

**C**

**B**

**A**

**
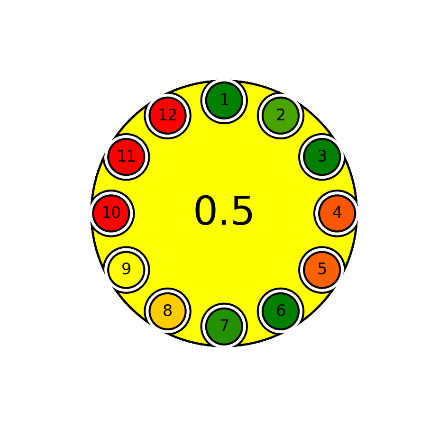

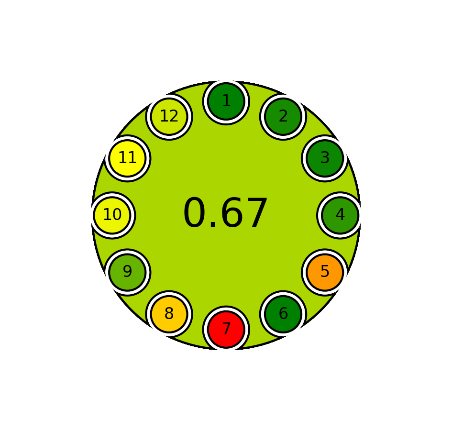

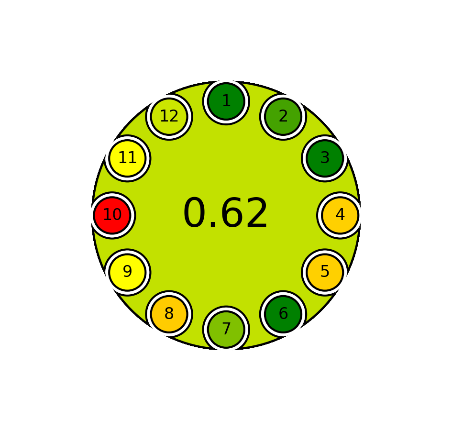
**

**E**

**D**

**
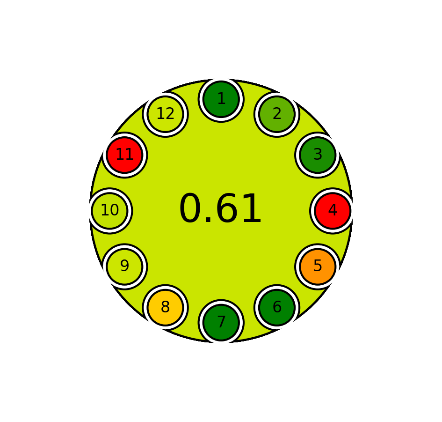

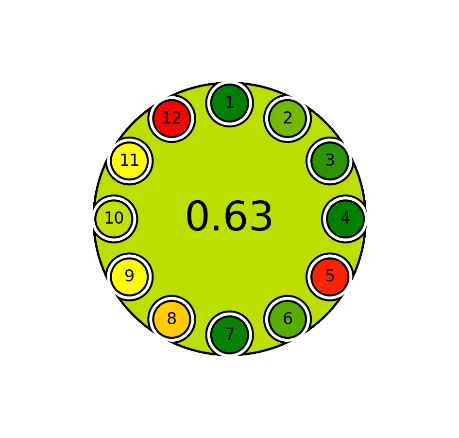
**

**Figure S15. AGREEmip evaluation of five DEX MIP workflows.** (A) Liu et al., 2017, (B) Du et al., 2018, (C) Hang et al., 2022, (D) Adauto et al., 2025, and (E) this study (MMIP10). The 12 radial segments represent the AGREEmip criteria: (1) removal of polymerization inhibitors, (2) functional monomer mass, (3) template mass, (4) cross-linking agent mass, (5) porogen/solvent mass, (6) other reagents or carriers, (7) core or surface-modification reagents, (8) polymerization initiation conditions, (9) particle size after processing, (10) elution solvent hazards, (11) elution technique, and (12) reusability of the final product. Colors reflect the relative greenness of each criterion, with green indicating favorable alignment and red indicating poor alignment. AGREEmip provides 0–1 score that reflect the extent of alignment with green chemistry principles, with higher values indicating better greenness.^6^

**References**

1. Pratama, K. F., Manik, M. E. R., Rahayu, D. & Hasanah, A. N. Effect of the molecularly imprinted polymer component ratio on analytical performance. *Chem. Pharm. Bull.* **68**, 1013–1024 (2020).

2. Prat, D. *et al.* CHEM21 selection guide of classical- and less classical-solvents. *Green Chem.* **18**, 288–296 (2016).

3. Wojnowski, W., Tobiszewski, M., Pena-Pereira, F. & Psillakis, E. AGREEprep – Analytical greenness metric for sample preparation. *TrAC - Trends Anal. Chem.* **149**, 116553 (2022).

4. Cherlet, M., De Baere, S., Croubels, S. & De Backer, P. Quantitative determination of dexamethasone in bovine plasma and tissues by liquid chromatography-atmospheric pressure chemical ionization-tandem mass spectrometry to monitor residue depletion kinetics. *Anal. Chim. Acta* **529**, 361–369 (2005).

5. Xu, Q. Incorporating solid-phase extraction into compendial procedures for the determination of dexamethasone and impurities in low-dose drug products. *J. Pharm. Biomed. Anal.* **175**, 112773 (2019).

6. Marć, M., Wojnowski, W., Pena-Pereira, F., Tobiszewski, M. & Martín-Esteban, A. AGREEMIP: The Analytical Greenness Assessment Tool for Molecularly Imprinted Polymers Synthesis. *ACS Sustain. Chem. Eng.* **12**, 12516–12524 (2024).
